# *Sarcandra glabra* alleviates intestinal injury via modulating gut microbiota and suppressing the IL-6/SOCS3 signaling pathway

**DOI:** 10.1038/s41538-026-00968-4

**Published:** 2026-07-01

**Authors:** Shaowei Huang, Xiaomei Chi, Siting Song, Xinyi Gao, Dedian Yu, Wenbin Huang, Jiayi Wang, Lixia Chen, Hua Li

**Affiliations:** 1 Institute of Structural Pharmacology & TCM Chemical Biology, Fujian Key Laboratory of Chinese Materia Medica, College of Pharmacy, Fujian University of Traditional Chinese Medicine, Fuzhou, China; 2 Wuya College of Innovation, Key Laboratory of Structure- Based Drug Design & Discovery, Ministry of Education, Shenyang Pharmaceutical University, Shenyang, China

**Keywords:** Diseases, Gastroenterology, Microbiology

## Abstract

Ulcerative colitis (UC) is a refractory inflammatory bowel disease characterized by gut microbiota dysbiosis and mucosal barrier disruption. *Sarcandra glabra* (Sg), a traditional herbal tea and dietary supplement, has been widely used to alleviate intestinal inflammation; however, its gut microbiota-dependent mechanism remains insufficiently clarified. In this study, Sg at 2, 4, and 8 g/kg was found to dose-dependently mitigate dextran sulfate sodium (DSS)-induced colitis in mice, restore mucosal barrier integrity by upregulating tight junction proteins and enhancing mucin secretion, and reduce serum levels of IL-1β, TNF-α, and IL-6. Integrative analysis of full-length 16S rRNA gene sequencing, transcriptomics, and metabolomics data revealed that Sg reshaped gut microbiota, selectively enriching *Ligilactobacillus animalis* while depleting *Escherichia coli* and *Proteus vulgaris*. We identified 8 microbiota-derived metabolites correlated with *Ligilactobacillus animalis* abundance, among which 2,3-dihydroxybenzoic acid (2,3-DHBA) exhibited the strongest protective effect against DSS-induced NCM460 cell damage. Mechanistic studies indicated that 2,3-DHBA effectively inhibited the IL-6/SOCS3 pathway and restored tight junction protein expression, effects that were reversed by exogenous IL-6. These findings demonstrate that Sg enriches for *Ligilactobacillus animalis* and its metabolite 2,3-DHBA, which suppresses IL-6-driven inflammation and repairs mucosal damage, supporting its potential as a microbiome-targeted dietary adjunct for UC.

## Introduction

Ulcerative colitis (UC) is a chronic recurrent condition marked by widespread inflammation of the colonic mucosa, with clinical manifestations including persistent diarrhea, mucus-containing stools, and bloody stools, severely affecting patients’ quality of life^1^. It is currently believed that the pathophysiology of UC entails complex interactions among environmental factors, immune system dysfunction, genetic susceptibility, and dysbiosis of the gut microbiota^2^. Among these, compromised gut barrier integrity is a pivotal factor in the pathogenesis of UC^3^. Barrier dysfunction leads to the translocation of antigens from the intestinal lumen into the submucosa, which, in turn, abnormally activates immune cells, resulting in the release of large amounts of inflammatory mediators, including IL-6 and TNF-α^4^. These inflammatory mediators further exacerbate intestinal epithelial damage, forming a vicious cycle of “barrier destruction-inflammation cascade” that constitutes the fundamental pathological underpinning of the persistent non-healing of UC^5^.

Regarding the above pathological processes, conventional clinical drugs such as aminosalicylates, glucocorticoids, and immunosuppressants can effectively alleviate acute symptoms, but their long-term use is often associated with significant toxic side effects and a very high risk of recurrence. In recent years, although JAK inhibitors and anti-TNF-α monoclonal antibodies have shown efficacy, their high prices and potential risk of systemic infections have limited their widespread use as first-line drugs^6,7^. These constraints have intensified interest in identifying safe, mechanism-defined alternatives from botanicals with established dietary or traditional use.

*Sarcandra glabra* (Thunb.) Nakai (Sg), a traditional Chinese medicine (TCM), has long been used in Asia to manage inflammatory conditions, including acute enteritis and dysentery^8^. Beyond its medicinal applications, Sg is consumed as an herbal tea and dietary supplement, reflecting its dual identity bridging traditional therapeutics and dietary practice^9^. Accumulating pharmacological evidence has validated that Sg contains bioactive compounds, including sesquiterpenes, flavonoids, and phenolic acids, with significant anti-inflammatory, antibacterial, and immune-regulatory properties^10–12^. Although Sg has shown strong intestinal protective potential in clinical applications, its precise therapeutic mechanisms in treating UC, particularly its modulation of the interaction between the gut microenvironment and host immunity, remain poorly understood.

Recently, the gut microbiota has been regarded as an important metabolic organ that maintains host homeostasis^13^. Extensive research has demonstrated significant dysbiosis in UC patients, while the successful clinical practice of fecal microbiota transplantation (FMT) highlights the central role of microbiome regulation in UC treatment^14,15^. With its multi‑component and multi‑target characteristics, TCM offers unique advantages for reshaping microbial communities and promoting the production of functional metabolites^16,17^. Regulation of the microbiota-metabolite-host axis has become a key pathway in modern TCM research. However, whether Sg can antagonize host pro-inflammatory signaling pathways by directing the enrichment of specific probiotics and producing key metabolites remains to be further explored.

Based on this, our study employed a DSS-induced acute colitis mouse model, integrating full-length 16S rRNA gene sequencing, transcriptomics, and non-targeted metabolomics to systematically evaluate the therapeutic effects of Sg on UC. We elucidate its mechanisms of action at multiple levels, including the protection of the intestinal barrier, the suppression of inflammatory signaling, and modulation of the gut microbiota, with a specific emphasis on the mediating role of microbiota-derived metabolites. These results provide a theoretical basis for the traditional clinical application of Sg and suggest its potential as a microbiota-targeted therapeutic dietary supplement for UC.

## Results

### Chemical composition and batch stability evaluation of Sg

Using UHPLC-QE-MS non-targeted metabolomics to analyze Sg components, we identified 806 chemical constituents. These primarily included terpenoids (276 types), phenolic acids (116), alkaloids (107), flavonoids (102), lignans and coumarins (63), steroids (6), quinones (5), tannins (3), and other classes (128) (Supplementary Fig. S1).

**Fig. 1:**
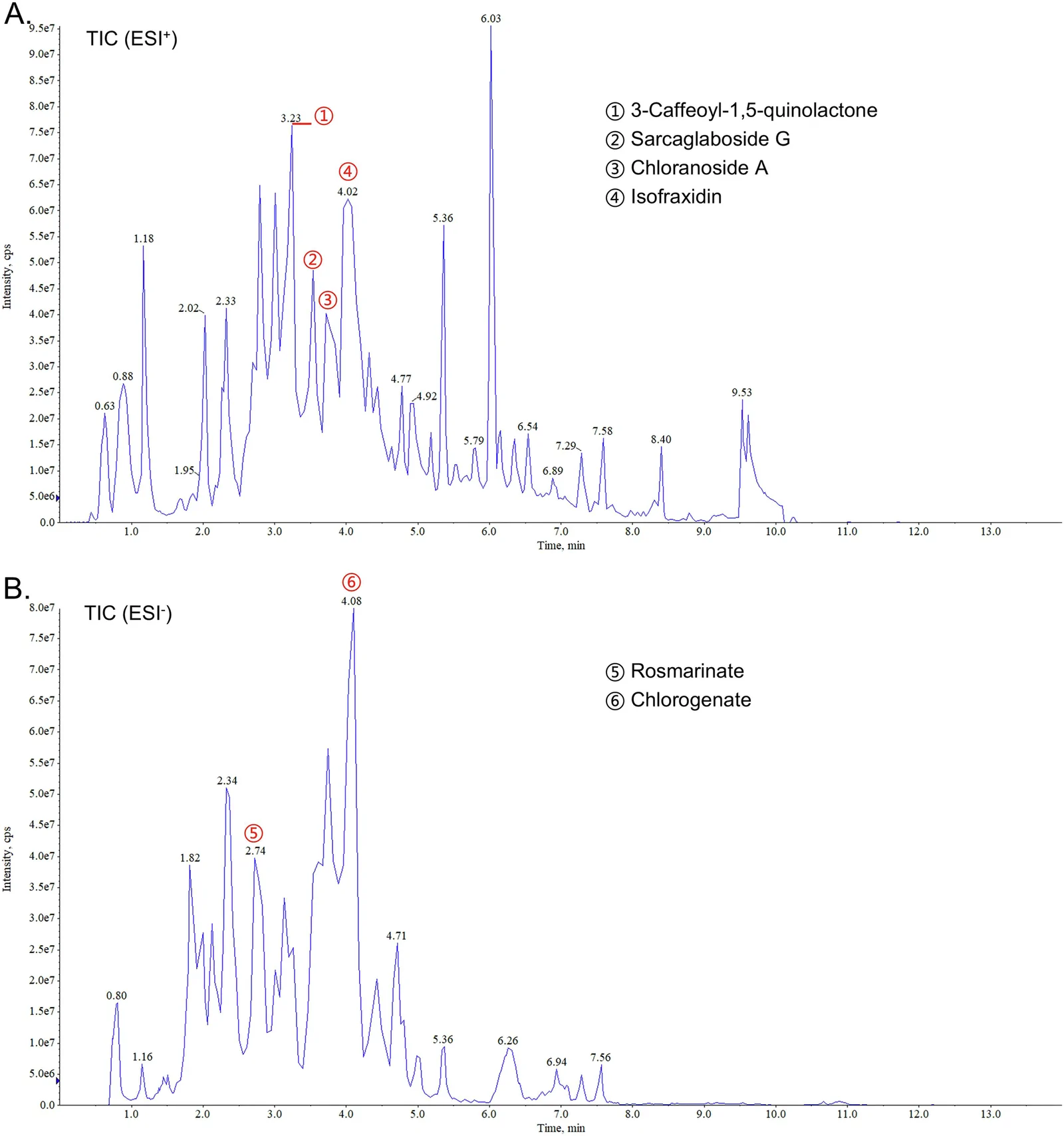
Identification of the chemical composition of Sg. **A** Positive ion mode; **B** Negative ion mode. ① 3-Caffeoyl-1,5-quinolactone; ② Sarcaglaboside G; ③ Chloranoside A; ④ Isofraxidin; ⑤ Rosmarinate; ⑥ Chlorogenate.

Six representative phytochemical markers were selected for stability assessment, based on the rationale that consistent detection across batches ensures reproducibility, regardless of their relative content. These markers—3-Caffeoyl-1,5-quinolactone, Sarcaglaboside G, Chloranoside A, Isofraxidin, Rosmarinate, and Chlorogenate (Fig. 1)—showed stable and consistent levels across batches (Supplementary Fig. S2). This indicates good batch consistency, providing quality assurance for pharmacodynamic studies.

### Sg ameliorates clinical symptoms in colitic mice

**Fig. 2:**
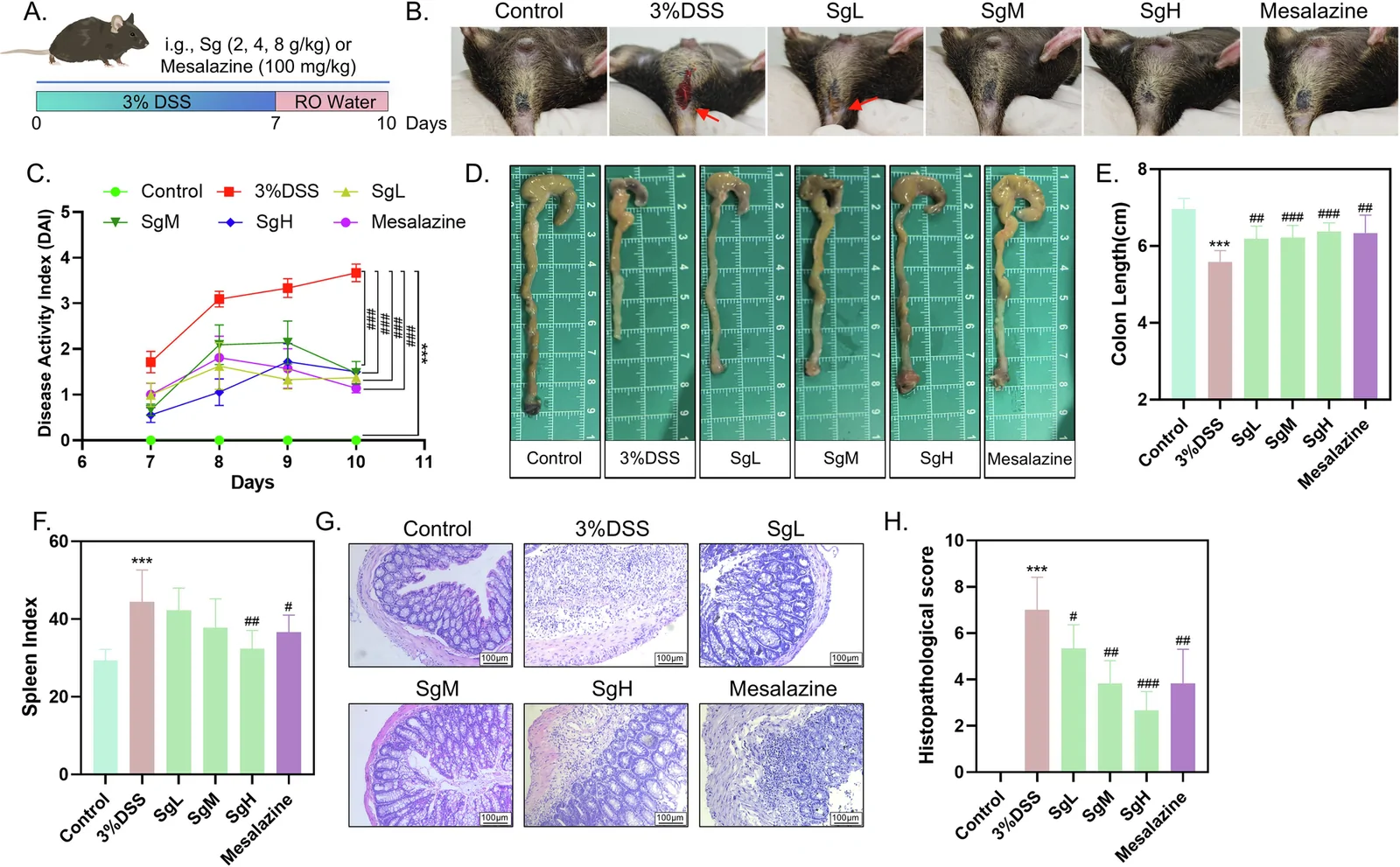
Sg ameliorates clinical symptoms in DSS-induced colitic mice. **A** Establishment of a mouse model of colitis and the administration method; **B** Anus bleeding situation; **C** Disease Activity Index (DAI) score; **D** Representative images of each group of colons; **E** Colon length; **F** Spleen Index; **G** Representative images of HE staining of the colon in each group (200×); **H** Colon tissue pathological score. *n* = 6. SgL, Sg low dose (2 g/kg) group; SgM, Sg medium dose (4 g/kg) group; SgH, Sg high dose (8 g/kg) group. *vs*. Control, ***P <0.001; *vs*. 3%DSS, ^#^*P* <0.05, ^##^*P* <0.01, ^###^*P* <0.001.

To evaluate Sg’s therapeutic effect on UC, an acute colitis mouse model was established using 3% DSS (Fig. 2A). As shown in Fig. 2B, three doses of Sg alleviated fecal bleeding in model mice. DAI was markedly upregulated in the 3% DSS compared with the control, confirming the successful establishment of colitis. Following Sg intervention, DAI scores decreased significantly across all dose groups, with the high-dose group approaching the positive control mesalazine group by day 10 (Fig. 2C). Additionally, Sg intervention significantly reduced colon shortening and splenomegaly in colitis mice (Fig. 2D–G), exhibiting a dose-dependent impact, with maximal efficacy noted in the high-dose group. Histopathological analysis revealed extensive inflammatory cell infiltration, intestinal epithelial cell sloughing, and loss of glandular architecture in the colon mucosa of colitic mice. All Sg dose groups significantly ameliorated these pathological lesions (Fig. 2G), with markedly reduced pathological scores. The effects of the Sg (4 and 8 g/kg) groups were analogous to those of the mesalazine group (Fig. 2H). These results indicate that Sg effectively ameliorates clinical symptoms of colitis.

### Sg repairs intestinal mucosal damage and suppresses inflammation in colitic mice

**Fig. 3:**
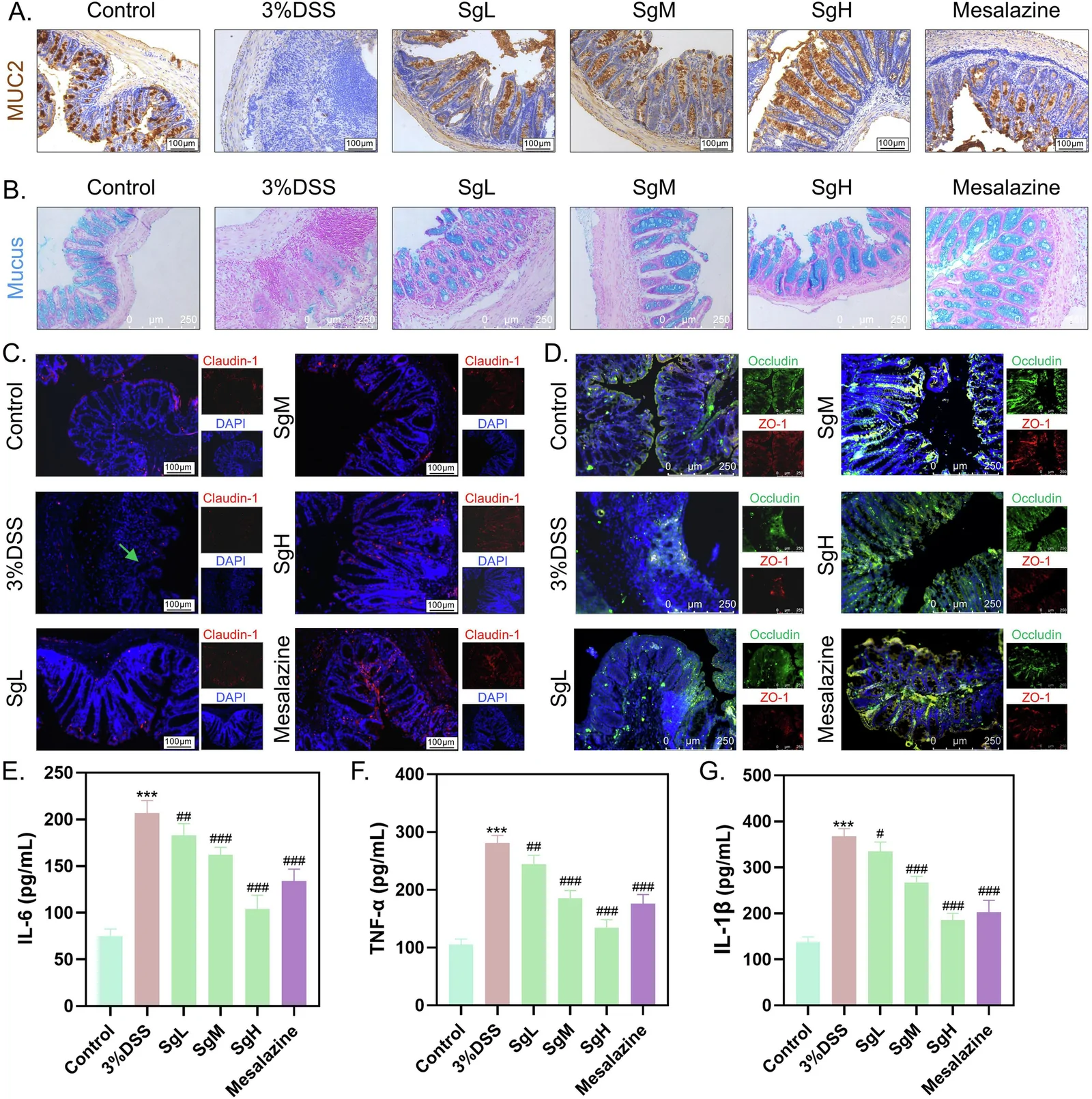
Sg repairs intestinal mucosal damage and suppresses inflammation in colitis mice. **A** Expression of MUC2 in the colon (200×); **B** The content of colonic mucus (blue, 200×); **C** Expression of Claudin-1 (red) in the colon (200×); **D** Expression of ZO-1 (red) and occludin (green) in the colon (200×); **E** The content of IL-6 in the serum; **F** The content of TNF-α in the serum; **G** The content of IL-1β in the serum. *n* = 6. vs. Control, ***P <0.001; vs. 3%DSS, ^##^*P* <0.01, ^###^*P* <0.001.

For prolonged UC, gut barrier dysfunction is a major pathophysiological basis. This study first examined changes in mucin MUC2 and mucus expression, critical indicators of the intestinal chemical barrier. Immunohistochemistry revealed significantly downregulated MUC2 protein expression in the colons of 3% DSS-treated mice, with complete disappearance in areas of glandular loss. Alcian blue staining revealed that mucus (blue) was only sparsely expressed in residual glandular areas, showing significant differences compared to the control group (Fig. 3A, B). Notably, low, medium, and high doses of Sg all restored MUC2 and mucus expression levels, with the effects of medium and high doses comparable to those of mesalazine. Immunofluorescence examination demonstrated markedly elevated expression of TJ proteins in all Sg dose groups relative to the 3% DSS group (Fig. 3C, D), suggesting that Sg enhances intercellular junction integrity in intestinal epithelial cells. Intestinal barrier disruption results in transintestinal antigen translocation, activating the innate immune system and releasing proinflammatory factors, forming a vicious cycle of “barrier damage-inflammation”. ELISA results showed that Sg dose-dependently reduced levels of IL-1β, TNF-α, and IL-6 in colitic mice (Fig. 3E–G). In summary, Sg exerts therapeutic effects by repairing intestinal mucosal barrier damage and suppressing intestinal inflammatory responses.

### Sg inhibits the IL-6/SOCS3 inflammatory signaling pathway in colitic mice

**Fig. 4:**
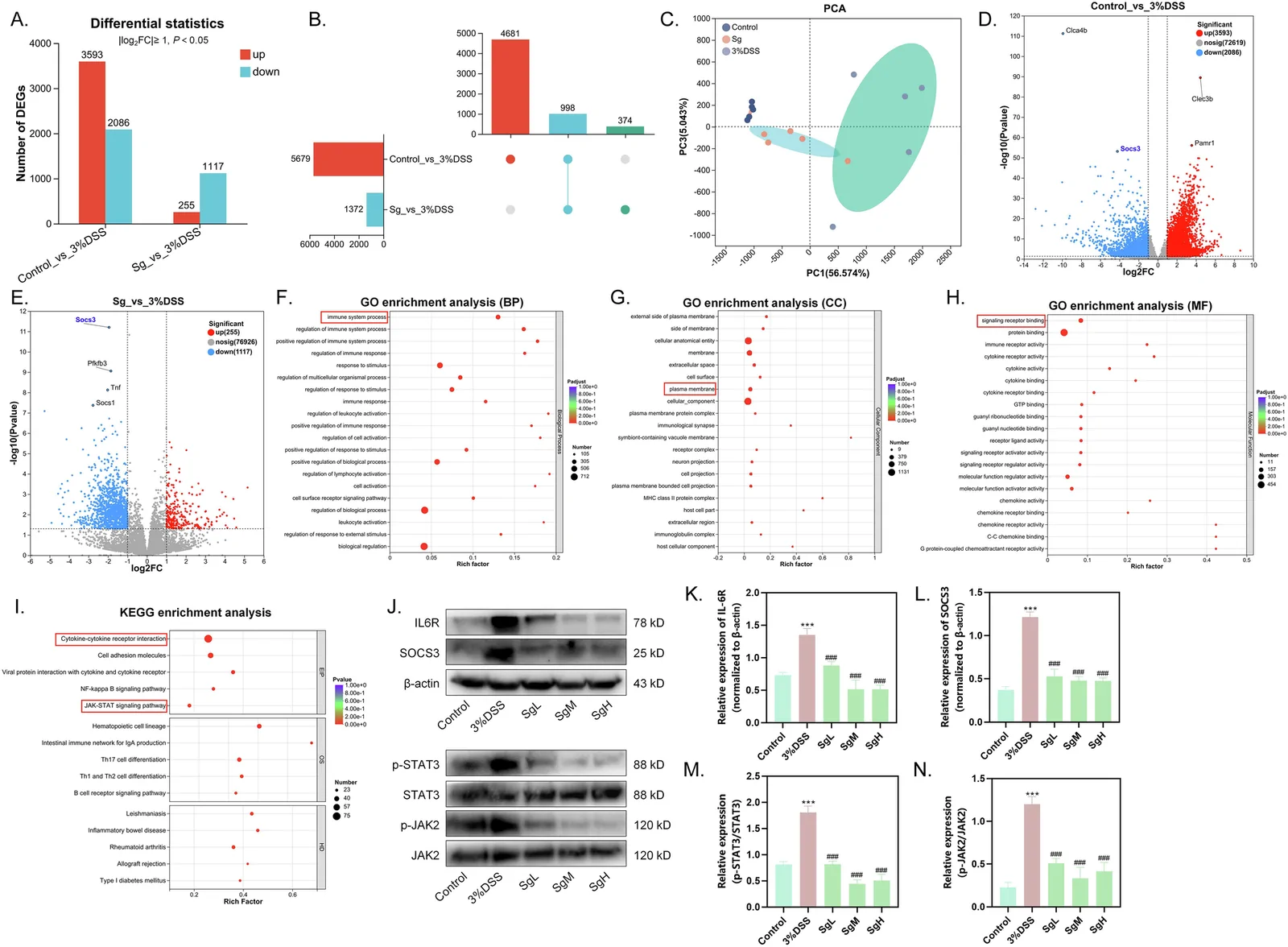
Sg inhibits the IL-6/SOCS3 inflammatory signaling pathway in colitis mice. **A** Transcriptome sequencing analysis of the differences in genes between the control group and the Sg group compared to 3% DSS group (|log2FC| ≥ 1, *P* <0.05); **B** The upset plot of the genes that are common to both Control vs 3%DSS and Sg vs 3%DSS; **C** PCA analysis in different groups; **D** Control vs 3% DSS volcano plot; **E** Sg vs 3%DSS volcano plot; **F** GO enrichment analysis (biological process) of the differentially expressed genes between Sg and 3% DSS; **G** GO enrichment analysis (cellular component); **H** GO enrichment analysis (molecular function); **I** KEGG enrichment analysis of the differentially expressed genes between Sg and 3% DSS; **J**–**N** The expression of IL-6R, SOCS3, p-JAK2, and p-STAT3 proteins in the colon. *n* = 5. Sg, Sg high dose (8 g/kg) group. *vs*. Control, ***P <0.001; *vs*. 3%DSS, ^###^*P* <0.001.

To elucidate the molecular mechanism of Sg’s gut mucosal barrier repair, transcriptomic sequencing analysis was performed on mouse colon tissues. In contrast to the control group, the 3% DSS group identified 2086 upregulated genes and 3593 downregulated genes; after Sg intervention, the number of differentially expressed genes decreased to 255 upregulated and 1117 downregulated genes (Fig. 4A). The Upset plot revealed 998 differentially expressed genes common to all three groups (Fig. 4B). PCA analysis indicated that gene expression profiles in the Sg group shifted toward the control group, suggesting that Sg partially reversed DSS-induced transcriptional disruption (Fig. 4C). Focusing on key regulatory genes, volcano plot analysis revealed SOCS3 as the most significantly co-downregulated gene in the intersection of the top five genes significantly downregulated in Control vs 3% DSS and Sg vs 3% DSS (Fig. 4D, E).

SOCS3, a critical negative feedback regulator in the JAK/STAT pathway, is crucial for preserving intestinal immunological homeostasis, with its expression primarily regulated by IL-6. KEGG analysis was predominantly enriched in “cytokine-cytokine receptor binding” and “JAK-STAT signaling pathway” (Fig. 4I). Additionally, GO analysis highlighted enrichment in “immune system process”, “plasma membrane”, and “signaling receptor binding” (Fig. 4F–H). Western blot validation confirmed that all doses of Sg significantly suppressed the expression of p-JAK2, IL-6R, SOCS3, and p-STAT3 proteins in the colon of colitis mice (Fig. 4J–N). Based on these findings, we hypothesize that Sg may alleviate intestinal inflammation and promote mucosal repair by attenuating the IL-6/SOCS3 pathway, thereby blocking excessive JAK/STAT activation.

### Sg restores gut microbiota homeostasis in colitic mice

**Fig. 5:**
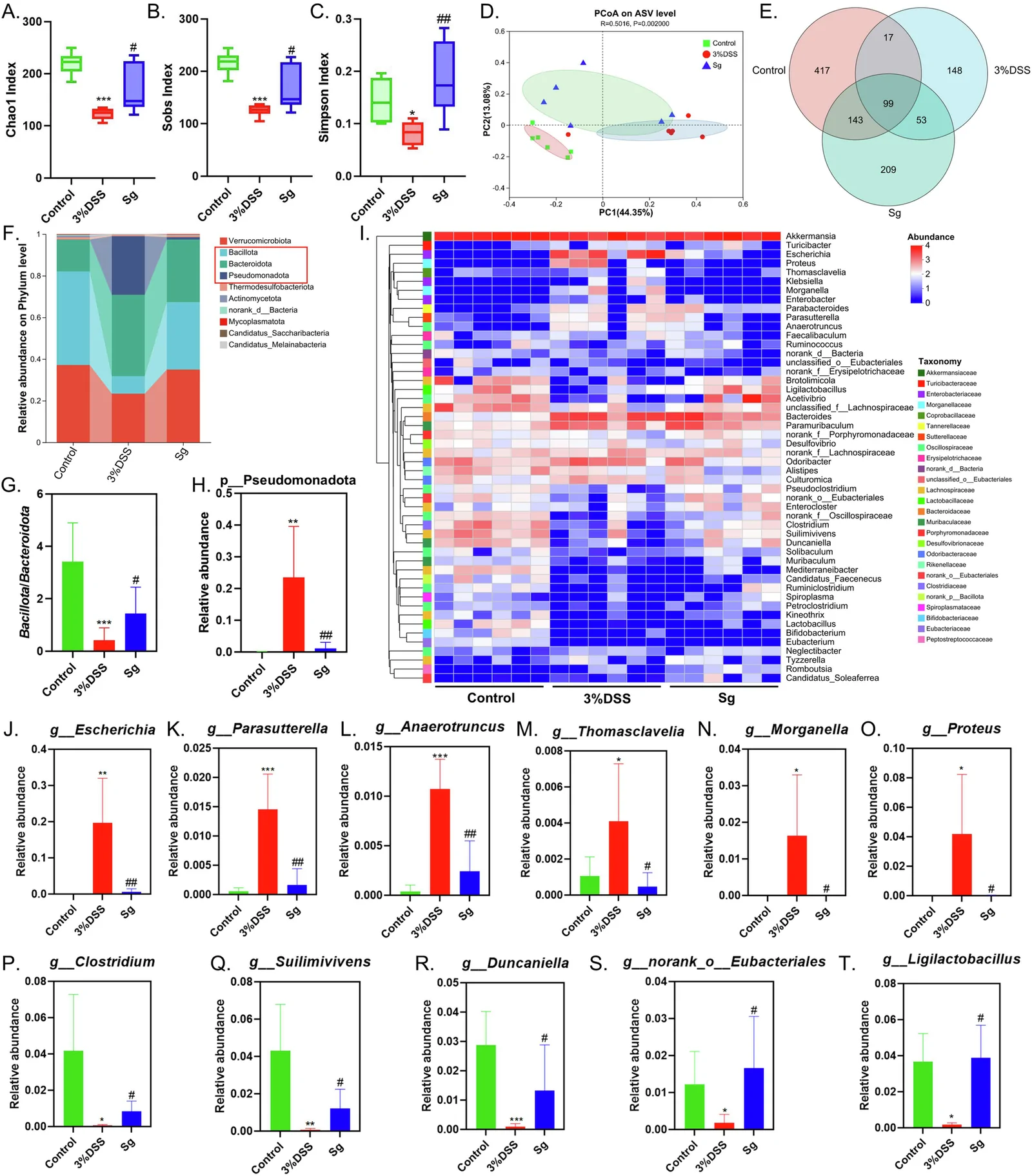
Sg restores gut microbiota homeostasis in colitic mice. **A** Chao1 index; **B** Sobs index; **C** Simpson index; **D** PCoA analysis of ASV in different groups; **E** The Venn diagram of the three groups; **F** Bar plot of community at phylum level; **G** The ratio of Bacillota/Bacteroidota in different groups; **H** The relative abundance of Pseudomonadota; **I** Heatmap of the top 50 bacterial genera with the highest relative abundance at the genus level; **J**–**T** The significant-difference bacterial genera among the top 50. *n* = 6. vs. Control, *P <0.05, **P <0.01, ***P <0.001; vs. 3%DSS, ^#^*P* <0.05.

Gut microbiota dysbiosis exacerbates intestinal mucosal damage by activating inflammatory signaling pathways. To characterize the regulatory effects of Sg on gut microbiota in colitic mice, full‑length 16S rRNA gene sequencing was performed on the high‑dose (8 g/kg) Sg group (the maximal‑effect dose chosen for mechanistic clarity). Analysis of α‑diversity revealed that Sg significantly restored the Chao1 index, Simpson index, and Sobs index of the gut microbiota in colitis (Fig. 5A–C). PCoA analysis indicated that the 3% DSS group was markedly distinct from the control group, while the Sg group clustered closely with the control group, suggesting that Sg regulates the microbiota structure toward normalization (Fig. 5D). Venn diagram analysis revealed 242 shared ASVs between the Sg and control groups, significantly exceeding the 116 shared ASVs between the 3% DSS and control groups, indicating that Sg mitigated DSS-induced loss of gut microbiota diversity (Fig. 5E). At the phylum level, Sg significantly restored the Bacillota/Bacteroidota ratio (*P* < 0.05; Fig. 5F, G) and markedly suppressed the abundance of the conditionally pathogenic phylum Pseudomonadota (Fig. 5H), consistent with previous studies on UC microbiota characteristics. At the genus level, differential analysis of the top 50 abundant genera revealed that Sg reduced the relative abundance of *Escherichia*, *Parasutterella*, *Morganella*, *Proteus* (all belonging to Pseudomonadota), *Anaerotruncus*, and *Thomasclavelia*, while increasing the relative abundance of *Clostridium*, *Suilimivivens*, *g__norank_o__Eubacteriales*, *Ligilactobacillus* (all belonging to Bacillota), and *Duncaniella* (Fig. 5I–T), corroborating the phylum-level findings.

**Fig. 6:**
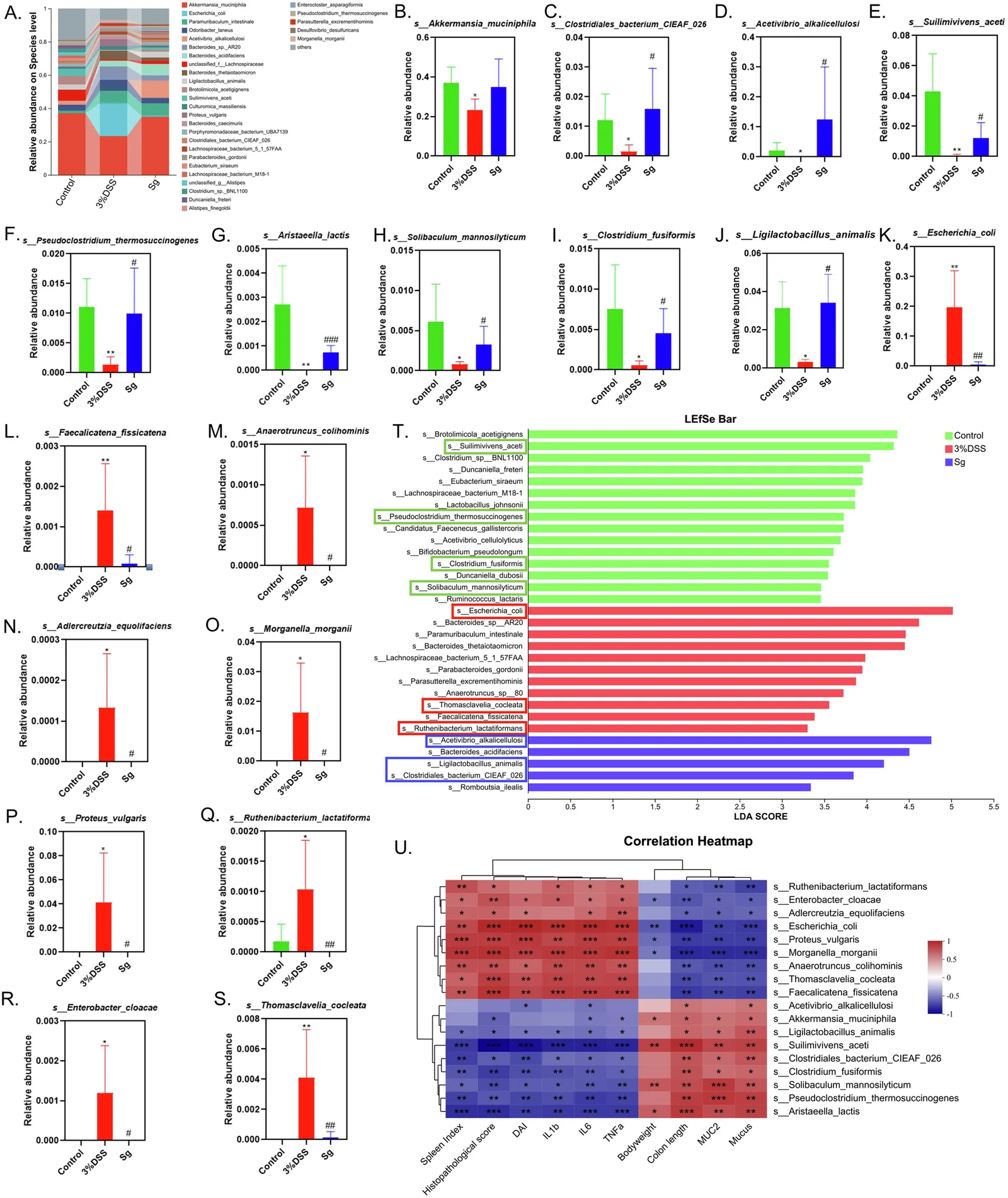
The correlation between the changes at the species level of gut microbiota and the efficacy of Sg. **A** Bar plot of community at species level; **B**–**S** Statistically significant species within the top 50 most abundant taxa; **T** LEfSe analysis at the species level; **U** Pearson correlation analysis between 18 differentially abundant species and pharmacodynamic indices. *n* = 6. vs. Control, *P <0.05, **P <0.01; vs. 3%DSS, ^#^*P* <0.05, ^##^*P* <0.01, ^###^*P* <0.001.

At the species level, high‑dose Sg (8 g/kg) significantly enriched nine bacterial species: *Akkermansia muciniphila*, *Clostridiales bacterium CIEAF_026*, *Acetivibrio_alkalicellulosi*, *Suilimivivens_aceti*, *Pseudoclostridium_thermosuccinogenes*, *Aristaeella_lactis*, *Solibaculum_mannosilyticum*, *Clostridium_fusiformis*, and *Ligilactobacillus_animalis* (Fig. 6A–J). Simultaneously, Sg suppressed nine bacterial species: *Escherichia coli*, *Faecalicatena fissicatena*, *Anaerotruncus colihominis*, *Adlercreutzia equolifaciens*, *Morganella morganii*, *Proteus vulgaris*, *Ruthenibacterium lactatiformans*, *Enterobacter cloacae*, *Thomasclavelia cocleata* (Fig. 6K–S). LEfSe analysis further validated that *Suilimivivens aceti*, *Pseudoclostridium thermosuccinogenes*, *Solibaculum mannosilyticum*, and *Clostridium fusiformis* were preferentially enriched in the Sg‑treated and control groups, whereas *Escherichia coli*, *Ruthenibacterium lactatiformans*, and *Thomasclavelia cocleata* were markedly elevated in the DSS‑induced colitis group (Fig. 6T). Of note, *Acetivibrio alkalicellulosi*, *Clostridium fusiformis*, and *Ligilactobacillus animalis* ranked among the top 5 most abundant species in the Sg group. Pearson correlation analysis revealed that the nine Sg-enriched species showed negative correlations with DAI, pathological score, spleen index, and inflammatory cytokines, while exhibiting positive correlations with MUC2, body weight, mucus, and colon length. Conversely, the nine inhibited species demonstrated opposite correlation patterns (Fig. 6U). These data indicate that Sg may mitigate colitis by correcting gut microbiota imbalance through a dual-regulatory strategy that promotes beneficial bacteria and suppresses pathogenic bacteria.

### Sg directly enriches beneficial bacteria *Ligilactobacillus animalis* and modulates microbiome metabolic function

**Fig. 7:**
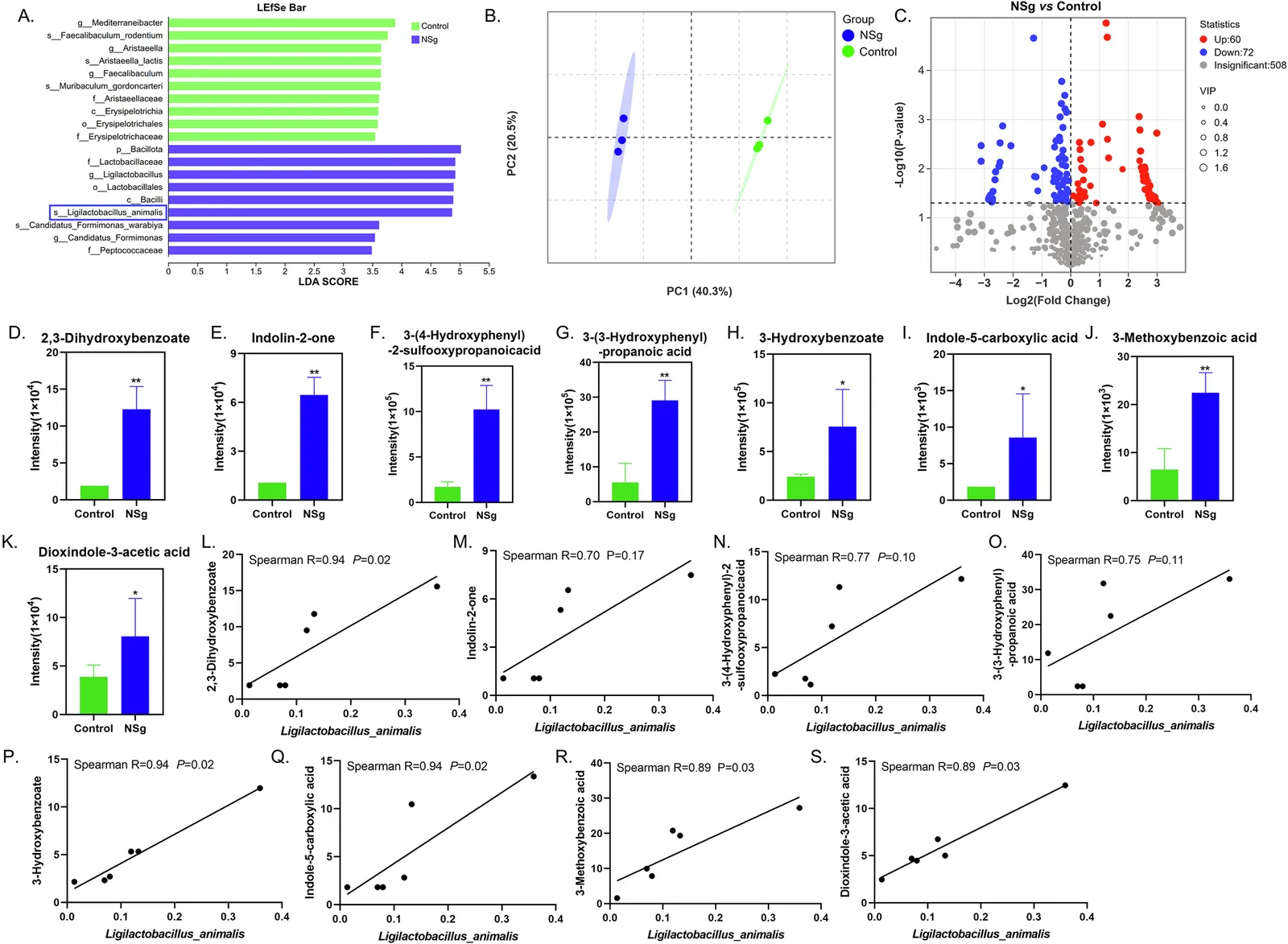
Sg directly enriches beneficial bacteria *Ligilactobacillus animalis* and modulates microbiome metabolic function. **A** LEfSe analysis of the gut microbiota in normal mice after Sg intervention; **B** PCA analysis of non-targeted metabolomics of Sg-containing serum (NSg) and control serum; **C** Control vs NSg volcano plot; **D**–**K** Bar plot of 8 upregulated gut microbiota-derived metabolites in the NSg group; **L**–**S** Spearman correlation analysis between 8 upregulated gut microbiota-derived metabolites and *Ligilactobacillus animalis*. n = 3. *vs*. Control, *P <0.05, **P <0.01.

To investigate whether Sg directly promotes beneficial bacteria in the gut independently of colitis resolution, we treated healthy wild‑type mice with Sg (NSg group). Results showed that Sg exerted no substantial impact on body weight, colon length, spleen index, or thymus index in normal mice (Supplementary Fig. S3A–F), indicating no overt disturbance to normal physiological homeostasis. 16S rRNA results revealed no substantial variations in Shannon index or PCoA analysis between the NSg group and the control group, suggesting limited effects of Sg on α-diversity and β-diversity of the normal mouse microbiota. Notably, LEfSe analysis revealed that Sg significantly enriched Bacillota, *Ligilactobacillus*, and *Ligilactobacillus animalis* in normal mice (Supplementary Fig. S3H–N, Fig. 7A). *Ligilactobacillus animalis* is a known beneficial bacterium that maintains intestinal barrier integrity and inhibits IL-6 expression, suggesting Sg may exert barrier protection by directly enriching this strain.

Further non-targeted metabolomics analysis of serum metabolites in NSg mice revealed 60 significantly upregulated and 72 significantly downregulated metabolites compared with the control group (Fig. 7B, C). Among these, eight upregulated metabolites were identified as gut microbiota-derived metabolites: 2,3-Dihydroxybenzoate (2,3-DHBA), Indolin-2-one (IO), 3-(4-Hydroxyphenyl)-2-sulfooxypropanoic acid (HPSPA), 3-Hydroxybenzoate (3-HBA), 3-(3-Hydroxyphenyl)-propanoic acid (3-HPPA), Indole-5-carboxylic acid (I5CA), 3-Methoxybenzoic acid (3-MBA), and Dioxindole-3-acetic acid (DI3AA) (Fig. 7D–K). Spearman correlation analysis revealed that *Ligilactobacillus animalis* exhibited positive correlations with all eight metabolites, with statistically significant associations observed for 2,3-DHBA, 3-HBA, I5CA, 3-MBA, and DI3AA (Fig. 7L-S).

### Sg alleviates colitis by inhibiting the IL-6/SOCS3 signaling pathway through microbiota-host interactions

**Fig. 8:**
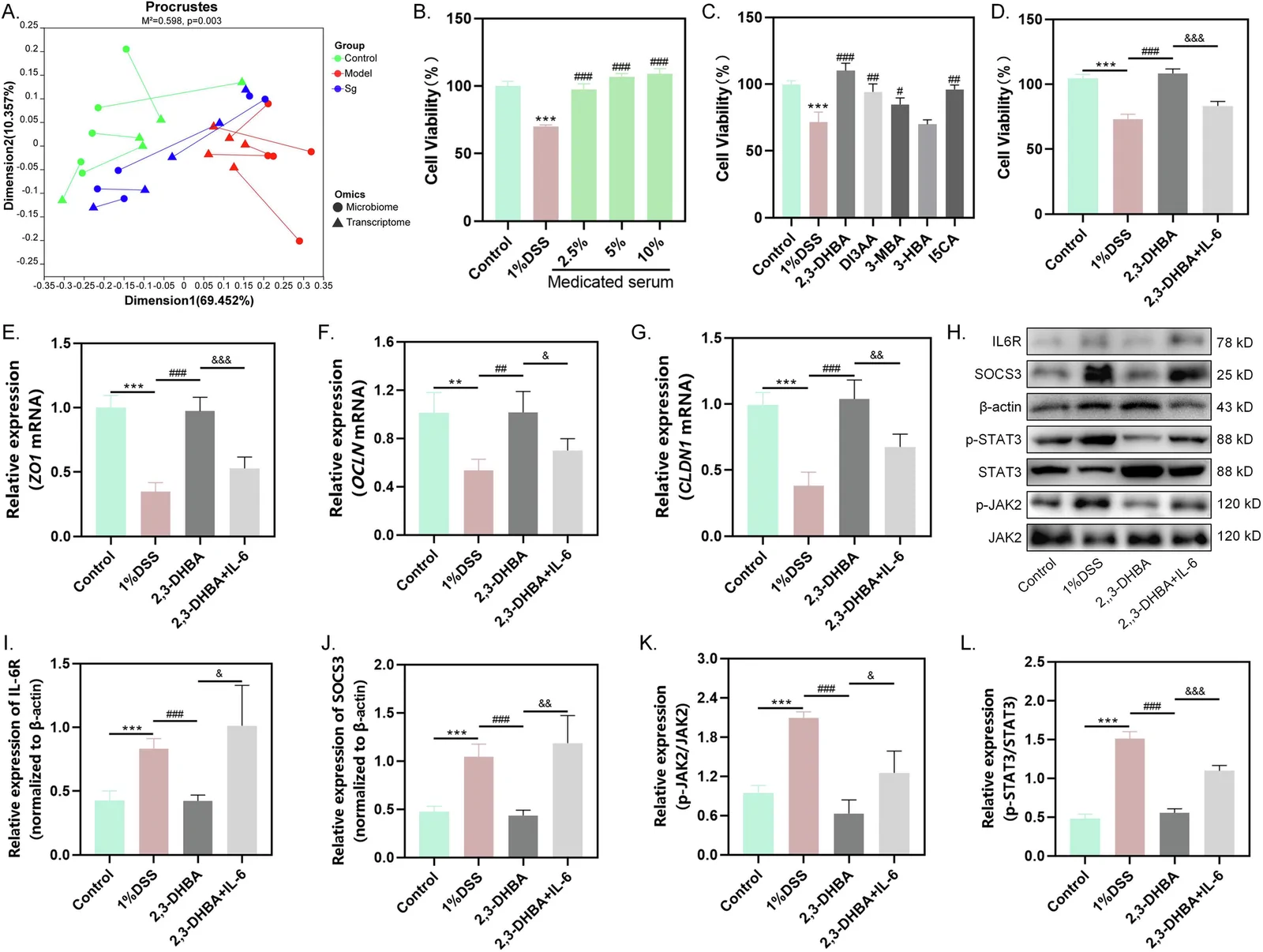
Metabolites derived from the microbiota repair DSS-induced damage to NCM460 cells by inhibiting the IL-6/SOCS3 signaling pathway. **A** Procrustes analysis of transcriptome and 16S rRNA gene sequencing; **B** Viability assessment of NCM460 cells exposed to 1% DSS following Sg-containing serum intervention; **C** Viability assessment of NCM460 cells exposed to 1% DSS following metabolites intervention; **D** Detection of cell viability in NCM460 cells treated with 2,3-DHBA in combination with or without the intervention of recombinant factor IL-6; **E**–**G** The mRNA expression levels of *ZO-1*, *OCLN*, and *CLDN1*; **H**–**L** The expression of IL-6R, SOCS3, p-JAK2, and p-STAT3 proteins in cells. *n* = 3. *vs*. Control, **P <0.01, ***P <0.001; *vs*. 1%DSS, ^##^*P* <0.01, ^###^*P* <0.001; *vs*. 2,3-DHBA, ^&^*P* <0.05, ^& &^*P* <0.01, ^& & &^*P* <0.001.

To explore the functional linkage between Sg‑mediated gut microbiota regulation and IL‑6/SOCS3 signaling inhibition, Procrustes analysis was performed using 16S rRNA gene sequencing and transcriptome data. The results indicated a substantial association between microbiome and transcriptomic data (M² = 0.598, *P* = 0.003), with the first principal component accounting for 69.45% of the variance (Fig. 8A). The three groups showed a tendency to separate at both the microbiome and transcriptome levels, with the Sg group positioned between the control and 3%DSS groups, suggesting that Sg intervention partially restores coordination between the microbiota and host transcription. In vitro experiments established an intestinal epithelial barrier-damage model using 1% DSS-treated NCM460 cells (Supplementary Fig. S4). Sg-containing serum intervention demonstrated effective restoration of cell viability at low (2.5%), medium (5%), and high (10%) concentrations (Fig. 8B). Further intervention with five metabolites significantly associated with *Ligilactobacillus animalis* (2,3-DHBA, 3-HBA, I5CA, 3-MBA, DI3AA) revealed that four metabolites (excluding 3-HBA) repaired barrier damage, with 2,3-DHBA demonstrating the strongest effect (Fig. 8C). Molecular docking results indicated that 2,3-DHBA had the lowest docking score, suggesting strong predicted binding affinity toward the IL-6/IL-6Rα/gp130 complex (Supplementary Fig. S5). Mechanism validation experiments demonstrated that 2,3-DHBA markedly upregulated tight junction *ZO-1*, *OCLN*, and *CLDN1* mRNA expression in NCM460 cells, while inhibiting the IL-6/SOCS3 signaling pathway. Notably, these protective effects were reversed by exogenous recombinant IL-6 (Fig. 8D–L). These findings demonstrate that 2,3-DHBA repairs intestinal mucosal barrier damage by preventing the IL-6/SOCS3 pathway. Integrating in vivo and in vitro results, we hypothesize that Sg may promote the production of metabolites such as 2,3-DHBA by enriching the beneficial gut bacterium *Ligilactobacillus animalis*, thereby suppressing the IL-6/SOCS3 pathway and ultimately repairing the intestinal mucosal barrier and alleviating inflammation.

## Discussion

UC, a clinically refractory, recurrent, chronic inflammatory condition, arises from complex interactions among genetic predisposition, barrier dysfunction, immune dysregulation, and dysbiosis. Although aminosalicylate drugs remain the current first-line therapy, their efficacy is limited by treatment bottlenecks and associated systemic side effects. This study reveals that Sg exerts multi-level protective effects in DSS-induced UC models by reshaping the gut microbiota and suppressing the IL-6/SOCS3 pathway, providing new experimental evidence for the clinical utilization of Sg.

Research indicates that the intestinal barrier serves as the primary defense for maintaining gut homeostasis, with its disruption constituting a core pathological mechanism of UC^18^. The degradation of TJ proteins and defects in the mucus layer collectively underlie barrier disruption^19^. Existing studies predominantly focus on protective strategies targeting single barrier components, such as recombinant MUC2 protein or tight junction activators, yet their clinical translation outcomes remain limited. Our study reveals that Sg simultaneously restores both the chemical barrier (MUC2, mucus) and the mechanical barrier (ZO-1, Claudin-1, Occludin). This synergistic protective impact suggests that Sg may act on upstream regulatory mechanisms that modulate barrier damage. Further transcriptomic analysis narrowed the mechanism to the IL-6/SOCS3 pathway. In the pathological state of UC, the IL-6 signaling pathway is excessively activated: IL-6 associates with either the membrane-bound or soluble IL-6 receptor (IL-6R), recruiting and dimerizing gp130. This initiates a phosphorylation cascade in the downstream JAK2/STAT3 pathway, driving the transcription of pro-inflammatory genes and aggravating intestinal barrier impairment^20,21^. Notably, SOCS3, a classic negative feedback regulator of the JAK2/STAT3 pathway, is often overinduced in chronic inflammatory environments due to sustained stimulation by IL-6 and other signals^22,23^. This creates a hazardous “negative feedback failure” cycle that paradoxically exacerbates signaling dysregulation^24^. In this study, Sg downregulated SOCS3 expression while significantly reducing IL-6R and phosphorylated JAK2/STAT3 levels, indicating that it does not merely enhance negative feedback but also suppresses excessive IL-6 activation at the source. This mechanism aligns with recent therapeutic explorations targeting IL-6R monoclonal antibodies (e.g., Tocilizumab) in inflammatory bowel disease^25^. As a natural multicomponent system, Sg may achieve milder immunomodulation through synergistic multi-component interactions, potentially circumventing infection risks and resistance issues associated with biologics.

The causal relationship between gut dysbiosis and UC has been extensively documented, yet existing studies predominantly focus on descriptive analyses, lacking in-depth insights into the molecular mediators of microbiota-host interactions. Our study reveals that Sg achieves therapeutic effects by promoting the enrichment of *Ligilactobacillus animalis* and its strongly correlated metabolite 2,3-DHBA. As a key member of the *Lactobacillus* genus, previous research on *Ligilactobacillus animalis* has primarily focused on its acid-producing capacity and pathogen-suppressing functions^26,27^. This study directly links it to the control of the IL-6 pathway, expanding the functional understanding of this strain in intestinal immune modulation. Moreover, the abundance of *Ligilactobacillus animalis* exhibited a notable negative connection with serum IL-6 concentrations (Fig. 6U) and was also significantly correlated with pharmacodynamic indicators of Sg (DAI score, histopathology score, etc.). This further indicates that *Ligilactobacillus animalis* may alleviate colitis and suppress IL-6 expression, consistent with previous studies^28^. Simultaneously, 2,3-DHBA, a phenolic acid metabolite produced by gut microbiota, has historically been regarded as an intermediate in host-microbiota co-metabolism, with its direct biological activity receiving limited attention^29^. Molecular docking results revealed that 2,3-DHBA forms hydrogen bond interactions with key residues SER181, VAL175, and SER177 within the IL-6/IL-6R/gp130 complex, exhibiting the strongest binding affinity among five metabolites significantly positively correlated with *Ligilactobacillus animalis*. Moreover, studies have demonstrated that 2,3-DHBA can inhibit the increased secretion of IL-6 stimulated by bacterial lipopolysaccharides (LPS)^30^. Functional experiments further confirmed that the repair effect of 2,3-DHBA on DSS-induced damage in NCM460 cells could be reversed by exogenous recombinant IL-6, indicating its protective role in the intestinal mucosa by inhibiting the IL-6/SOCS3 pathway. Existing studies have also confirmed that 2,3-DHBA derived from microorganisms can effectively alleviate the inflammatory symptoms in DSS-induced UC mice^31^.

**Fig. 9:**
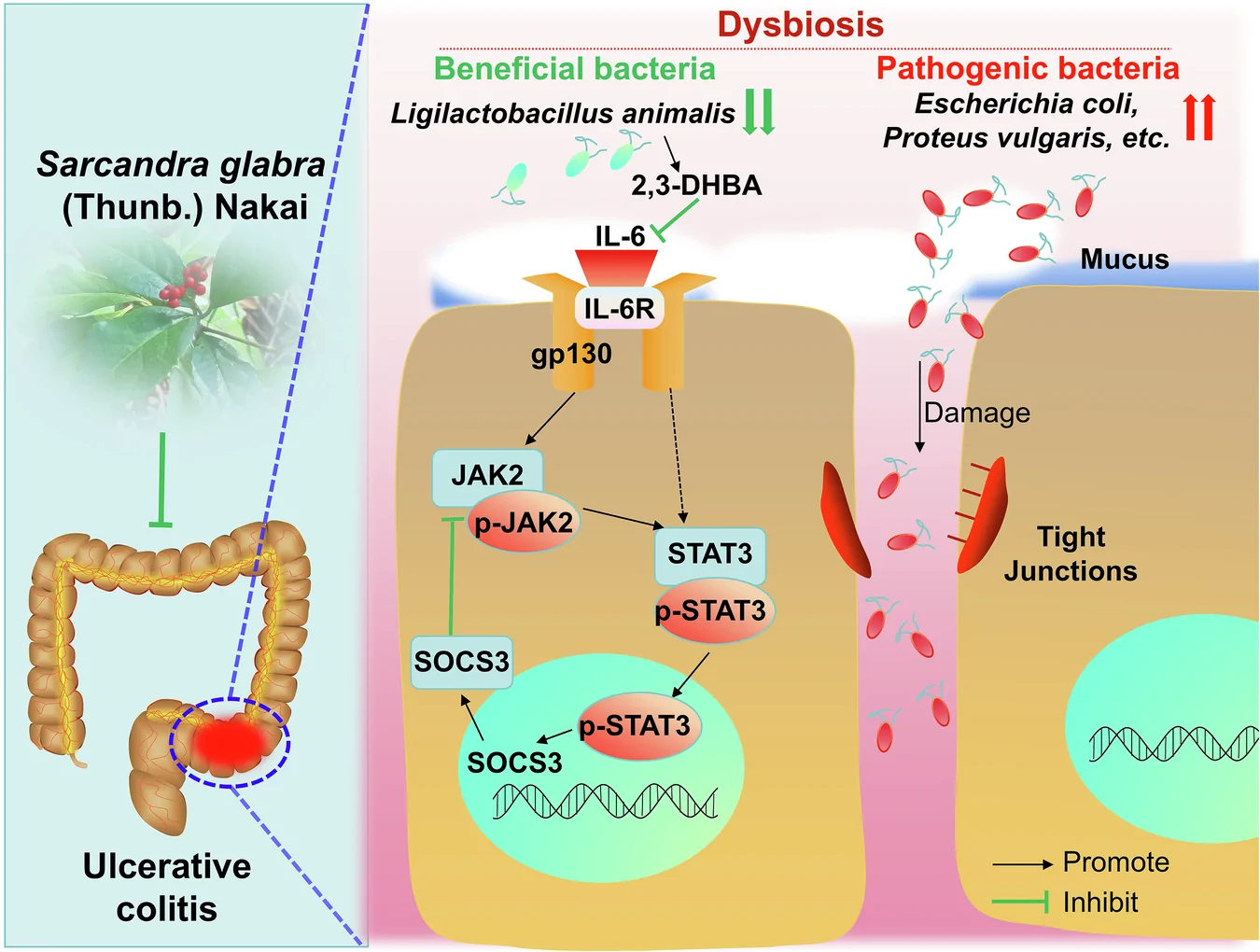
Sg alleviates intestinal mucosal injury via modulating gut microbiota and suppressing the IL-6/SOCS3 signaling pathway.

Furthermore, the inhibitory effect of Sg on specific bacterial genera also holds significant pathological implications. The enrichment of *Escherichia coli* in colitis models may reflect niche occupation by opportunistic pathogens following deterioration of the intestinal microenvironment^32^. In the inflamed gut, disrupted redox potential and increased availability of oxygen and nitrates provide a selective growth advantage for facultative anaerobes such as *Escherichia coli*, allowing them to outcompete obligate anaerobes^32^. This expansion is often associated with the massive release of LPS and other pro-inflammatory mediators, which further exacerbate mucosal injury^33^. Sg’s suppression of *Escherichia coli* is not merely antibacterial; it more likely achieves optimized microbial structure through ecological mechanisms such as restoring competitive exclusion and improving intestinal redox potential. This dual regulation, promoting beneficial bacteria while suppressing opportunistic pathogens, embodies an ideal strategy for microbiome intervention. It also explains why Sg has a limited impact on the normal mouse microbiota (to avoid disruption of homeostasis) yet effectively corrects dysbiosis in pathological states. These findings suggest that Sg not only suppresses inflammatory signaling by enriching beneficial bacteria (*Ligilactobacillus animalis*) and metabolite (2,3-DHBA), but also creates a favorable microenvironment for mucosal repair by inhibiting opportunistic pathogens (Fig. 9). This multi-level regulatory network involving “microbiota-metabolites-host” further highlights the unique advantage of traditional Chinese medicine in treating complex chronic diseases through multi-targeted, multi-component approaches.

While this multi-level study demonstrates that Sg alleviates colitis by repairing the intestinal mucosal barrier via modulation of gut microbiota and inhibition of the IL-6/SOCS3 pathway, several limitations should be acknowledged. First, although multi-omics correlation analysis and in vitro experiments established the association between *Ligilactobacillus animalis* and 2,3-DHBA with anti-inflammatory efficacy, the causal role of *Ligilactobacillus animalis* and 2,3-DHBA in Sg’s therapeutic effects remains to be validated in germ-free animal models and FMT experiments. Second, the specific active compounds in Sg that promote the enrichment of *Ligilactobacillus* *animalis* remain unidentified. Although our study identified 806 chemical constituents, the complexity of this chemical profile makes it difficult to attribute biological activity to individual compounds. Rosmarinate and chlorogenic acid, both identified in Sg, have been reported to selectively promote the growth of *Lactobacillus* while inhibiting pathogenic bacteria^11,34^, suggesting that multiple components in Sg may work in concert to regulate the gut microbiota. However, linking specific Sg constituents to the selective enrichment of *Ligilactobacillus animalis* would require a systematic screening approach where individual chemical constituents are tested for their ability to promote *Ligilactobacillus animalis* growth (prebiotic effect) or inhibit pathobiont growth (antimicrobial effect) in vitro, followed by validation in vivo. Given the complexity of Sg’s chemical profile, such systematic screening was beyond the scope of the current proof-of-concept investigation, and future component-microbiome association screening is warranted. Additionally, the interaction between 2,3-DHBA and the IL-6/IL-6R/gp130 complex is currently supported only by molecular docking predictions, and the exogenous IL-6 reversal experiment only demonstrates pathway dependence without establishing direct binding; further validation using SPR, ITC, or CETSA is needed to verify the binding mode. Moreover, this study utilized an acute DSS-induced colitis model that primarily models chemical epithelial injury rather than the immune-mediated chronicity characteristic of human UC. The acute injury paradigm may overestimate the relative contribution of barrier repair and microbiota-dependent mechanisms to Sg’s therapeutic effects. Future validation using chronic colitis models (e.g., IL-10 knockout mice, repeated DSS cycles, or T-cell transfer models) would be essential to assess whether Sg’s microbiota-dependent mechanisms are equally effective in immune-mediated chronic UC. Furthermore, the high dose (8 g/kg) translates to a human equivalent dose slightly exceeding the conventional clinical dosage specified in the Chinese Pharmacopoeia, and was adopted solely for preclinical efficacy exploration; both the low and medium doses fall within the converted pharmacopoeial dosage range and were sufficient to ameliorate intestinal inflammation. This study evaluates therapeutic application rather than preventive supplementation. Despite these objective limitations, this study retains non-negligible research value and effectively addresses common shortcomings in the current field of herbal medicine research for colitis. At present, the majority of gut microbiota-related studies remain at the level of simple differential analysis, often merely describing the macroscopic changes in microbiota structure induced by drugs, without in-depth exploration of the cascade relationships among core functional species, microbial metabolic mediators, and host inflammatory pathways, making it difficult to elucidate the scientific connotation of the “multi-component, multi-target” nature of traditional Chinese medicine. Distinguished from conventional microbiota studies, our study included a healthy mouse intervention group, excluded the passive, secondary changes in the microbiota resulting from inflammation resolution, and clearly demonstrated that Sg can directly enrich *Ligilactobacillus animalis*, thereby proving that microbiota modulation is an active pharmacological effect rather than an epiphenomenon of treatment. Simultaneously, by integrating serum non-targeted metabolomics to screen characteristic microbiota-derived metabolites and combining in vitro cell reversal experiments to validate the functional mediating role of 2,3-DHBA, this study clearly elucidated the intrinsic linkage of the “bacterial species-metabolite-inflammatory pathway” axis. Therefore, this study conclusively demonstrates that gut microbiota is an important mediating factor for Sg’s anti-colitis efficacy, providing a complete and traceable mechanistic chain for understanding how traditional Chinese medicine relies on gut microecological regulation to modulate intestinal inflammation, and establishing a solid data foundation and theoretical basis for subsequent in-depth investigation of causal mechanisms and pharmacological substance screening.

In summary, our research indicates that Sg significantly alleviates colitis symptoms and systematically elucidates its specific mechanisms for repairing intestinal mucosal damage. Sg achieves this through bidirectional regulation of gut microbiota composition: on one hand, it reduces the abundance of multiple potentially harmful bacteria, including *Escherichia coli* and *Proteus vulgaris*, while enriching beneficial bacteria, such as *Ligilactobacillus animalis*, and promoting their production of 2,3-DHBA. This metabolite then interacts with the IL-6R complex to inhibit the IL-6/SOCS3 signaling pathway, ultimately establishing a multi-tiered protective effect: “modulating gut microbiota→ promoting metabolite production→ suppressing host inflammation”. This study provides experimental support for Sg’s clinical application in UC and presents a framework for exploring microecological regulatory mechanisms of therapeutic dietary supplements.

## Methods

### Preparation of Sg

Sg was acquired from Kangmei Pharmaceutical Co., Ltd. (Puning, China). 50 g of Sg was immersed in eight times its weight of distilled water for 30 min, subsequently heated for thirty minutes, and finally filtered. The residue was then boiled again with six times the volume of distilled water for thirty minutes. The two filtrates were amalgamated and concentrated under reduced pressure, ultimately obtaining a decoction of Sg (1 g/mL). At the same time, the chemical composition of Sg was examined using non-targeted UHPLC-QE-MS metabolomics (Wuhan Maiwei Metabolomics Biotechnology Co., Ltd.). Parallel testing was performed on three batches of Sg extracts to evaluate fingerprint similarity and decoction stability. Representative components covering the main chemical classes of Sg were selected for the stability assessment, not based on their relative content, but rather on their reliable and consistent detection across batches. The selection of active constituents in Sg was mainly guided by previous pharmacological and phytochemical studies^35,36^.

### Animals and treatments

Male C57BL/6 mice (18–22 g; Shanghai Slac Laboratory Animal Co., Ltd., China) were kept under specific pathogen-free conditions (12 h light/dark cycle, 24 ± 2 °C, 40–80% humidity). All experimental procedures strictly adhered to animal ethics guidelines and received approval from the Animal Ethics Committee of Fujian University of Traditional Chinese Medicine (Approval No.: FJTCM IACUC 2024266).

A colitis model was established using DSS (MP Biomedicals, CA, USA). 60 mice were randomly allocated into six groups (*n* = 10): control, 3% DSS, three doses of Sg (2, 4, and 8 g/kg), and the positive drug (Mesalazine, 100 mg/kg). The three Sg doses were strategically selected based on the following rationale: as documented in the Chinese Pharmacopoeia, Sg is clinically prescribed for adult patients as a water decoction at daily doses of 9–30 g; based on body surface area conversion (Km ratio of mice to humans = 3/37), this clinical dosage range corresponds to ~1.5–6.2 g/kg in mice for a 60 kg human^37^. Furthermore, a previous study reported that doses ranging from 4.5 to 9 g/kg could effectively ameliorate intestinal inflammation in colitic mice^12^. Therefore, the three doses adopted in this study were chosen to cover the equivalent range of traditional clinical dosages, with the highest dose slightly surpassing the converted upper limit to facilitate the identification of a definitive dose-response relationship. Mice in every other group—aside from the Control group—freely drank a 3% DSS solution from days 1–6 to induce colonic injury, switching to sterile distilled water from days 7 to 10. During modeling, each treatment group received daily timed oral administration of the corresponding drug dose. For DAI quantification, animals underwent daily weighing, along with characterization of enteric discharge and gastrointestinal hemorrhage. Mice were euthanized on day 11. Spleens and colons were promptly dissected and isolated. Spleen weight was measured to calculate the spleen index, and the colon’s normal length (cm) was recorded.

Preparation of Sg-containing serum: C57BL/6 mice were allocated into a control group and an Sg (8 g/kg) group (NSg), with 6 mice per group. Drugs were administered via oral gavage once in the morning and once in the evening for 7 consecutive days. One hour post-administration of the final dose, blood was obtained through the ocular venous plexus. Blood was permitted to equilibrate at ambient temperature for an hour, then centrifuged for 15 min at 4 °C and 3000 rpm. The serum was harvested and preserved at −80 °C. Subsequently, the composition of Sg-containing serum was analyzed using non-targeted UHPLC-QE-MS metabolomics (Wuhan Maiwei Metabolic Biotechnology Co., Ltd.).

### Hematoxylin-Eosin staining

On day 11, distal colon tissue (~1 cm) was harvested from mice and promptly placed in 4% paraformaldehyde solution for 24-h fixation. Then, tissue specimens underwent graded ethanol dehydration and paraffin embedding, followed by sectioning into 4-μm-thick slices. Following routine hematoxylin and eosin staining, morphological changes were examined under an optical microscope, including epithelial structural integrity, inflammatory cell infiltration, and crypt damage. Histopathological scores were assessed using a previously defined methodology^38^.

### Enzyme-linked immunosorbent assay (ELISA)

Quantitative measurements of serum IL-1β, TNF-α, and IL-6 concentrations were performed using the protocols of a commercial ELISA kit (Jiangsu Enzyme Immunoassay Industrial Co., Ltd., China).

### Immunohistochemistry (IHC) staining

Strictly following the operating guidelines of the IHC kit (Bioss, China), we performed in situ detection to examine the distribution and expression of MUC2 protein (Servicebio, China) in paraffin-embedded colon tissue sections.

### Immunofluorescence (IF) staining

After dewaxing colon paraffin sections in water, place them in sodium citrate buffer (pH 6.0) for 8 min to retrieve antigens. Allow to cool to room temperature naturally, then permeabilize with 0.3% Triton X-100 (0.5 h) to improve antibody infiltration. Subsequently, sections were treated (0.5 h) with 10% goat serum. In a humidified, light-protected place, ZO-1, Claudin-1, and Occludin antibodies (Proteintech, China) were incubated with sections for 14 h at 4 °C. On the subsequent day, Alexa Fluor 488-labeled or Cy3-labeled secondary antibodies were introduced, and sections were incubated at ambient temperature in the dark for 1 h. After thorough washing with PBS, slides were affixed using a DAPI-containing solution. Representative images were captured under a microscope with fluorescence to evaluate the distribution of tight junction (TJ) proteins.

### Alcian Blue-Nuclear Red staining

To evaluate colonic mucus secretion, this study employed the Alcian Blue-Nuclear Red staining kit (Beyotime, China) to stain paraffin-embedded colonic tissue sections. The manufacturer’s standard protocol was strictly followed: Alcian Blue was used for specific staining of acidic mucins, with Nuclear Red serving as a background counterstain. Following staining, colonic mucus expression was examined using an optical microscope.

### Full-length 16S rRNA gene sequencing

On Day 11, cecal contents were collected from the 3%DSS, Sg (8 g/kg), and control groups. After being quickly frozen in liquid nitrogen, the samples were moved to an ultra-low temperature freezer set (−80 °C) for storage. Total DNA from microbial communities, PCR, and sequencing library preparation were performed by Shanghai Meiji Biotechnology Co., Ltd (China). All bioinformatic analyses were conducted on the Meiji Bio Cloud Platform. Specifically, mothur software was used to calculate alpha diversity indices (Chao1, Sobs, Shannon), with Wilcoxon rank-sum tests applied for intergroup alpha diversity analysis. Finally, LEfSe analysis (Linear discriminant analysis Effect Size) was employed (screening criteria: LDA > 3, *P* < 0.05) to identify statistically significant distinctions in bacterial communities at the phylum-to-species level.

### RNA-sequencing analysis of colon tissue

On day 11, 1 cm of distal colon tissue was collected from control, 3%DSS, and high-dose Sg groups and promptly cryopreserved in liquid nitrogen. Total colon RNA, library preparation, and high-throughput sequencing were performed with assistance from Shanghai Meiji Biotechnology Co., Ltd. Differentially expressed genes (DEGs) were identified using raw data cleaning and alignment, with a threshold of |log₂FC| ≥ 1 and *P* < 0.05. Functional annotation via GO and KEGG pathway enrichment analysis was then integrated to focus on alterations in Sg-containing serum inflammatory signaling pathways.

### Cell culture and treatment

Normal human colonic epithelial cells (NCM460), sourced from Shanghai Zhongqiao Xinzhou Biotechnology Co., Ltd. (China), were maintained in complete DMEM medium (1% penicillin/streptomycin +10% FBS) (Gibco, USA) at 37 °C under 5% CO₂. To determine the optimal modeling concentration, the CCK-8 assay (APExBIO, USA) was employed to examine the impact of DSS (1%, 2%, 3%, 4%, 5%, 6%)-induced 22-hour treatment on NCM460 cell viability.

Sg-containing serum intervention: NCM460 cells were randomly allocated into the control (10% blank serum), 1% DSS (10% blank serum), and low-, medium-, and high-dose Sg drug-medicated serum groups. Each dose group received intervention with 2.5%, 5%, or 10% Sg-containing serum (total serum concentration adjusted to 10% with blank serum). After 2 h pretreatment, 1% DSS was added for co-incubation for 22 h. Cell viability was evaluated using the CCK-8 assay.

Key metabolite screening: The five metabolites 2,3-DHBA, 3-HBA, I5CA, 3-MBA, and DI3AA (5 μM) were used to pretreat the aforementioned model cells for 24 h to evaluate their protective effects against DSS-induced damage.

IL-6/SOCS3 signaling pathway recovery assay: To elucidate the target of 2,3-DHBA and its regulatory mechanism on the IL-6 signaling pathway, NCM460 cells were pretreated for 2 h in a solution containing 2,3-DHBA (5 μM) with or without recombinant IL-6 (10 ng/mL), followed by 22 h of continued culture with 1% DSS. Upon completion of the experiment, cell samples were harvested, cell viability was evaluated using the CCK-8 assay, tight junction mRNA expression was quantified by qRT-PCR, and phosphorylation levels and protein expression related to the IL-6/SOCS3 pathway were analyzed by Western Blot.

### Quantitative real-time polymerase chain reaction (qRT-PCR)

Total RNA was obtained from 60 mg of distal colon or cells utilizing TRIzol (Solarbio, China). The concentration and purity of RNA were evaluated using a spectrophotometer (NanoDrop 2000). Reverse transcription was conducted using 1 μg of total RNA as a template, adhering meticulously to the instructions provided by the reverse transcription kit to produce cDNA. Subsequently, RT-PCR was conducted utilizing cDNA as a template^39^, and the gene expression levels were assessed. All primer sequences^40^ involved in the experiment are detailed in Supplementary Table S1.

### Western blot

Colon or cell samples were thoroughly disrupted in RIPA lysis buffer (1% phosphatase inhibitor +1% PMSF) (Beyotime, China). Total protein was measured via the BCA approach, and 20 μg of protein sample was denatured in a boiling water bath before separation by SDS-PAGE electrophoresis. Proteins were then moved to PVDF membranes and treated for two hours at ambient temperature in 5% skim milk. Following the membrane trimming based on protein molecular weight, it was exposed overnight (4 °C) with designated antibodies against IL6R (abclonal, China), SOCS3 (servicebio, China), p-STAT3 (afantibody, China), STAT3 (afantibody, China), p-JAK2 (abclonal, China), and JAK2 (abclonal, China). The following day, after three washes with TBST buffer, the membranes were treated with the appropriate secondary antibodies for 1 h (ambient temperature). Following three supplementary TBST washes, protein bands were detected utilizing ECL Plus chemiluminescent substrate (Beyotime, China) and a chemiluminescence imaging system (Tanon, China). Finally, the target bands were evaluated with ImageJ and normalized against the internal control protein.

### Molecular docking

Molecular structures of 2,3-DHBA, 3-HBA, I5CA, 3-MBA, and DI3AA obtained from PubChem were combined with PyMOL and Autodock tools to construct and preprocess the IL-6R complex (PDB ID: 1P9M). Subsequently, the center of the grid box (height, width, and length) was defined. Autodock was used to perform molecular docking, and the results were shown in PyMOL for the analysis of interactions, such as hydrogen bonds and hydrophobic interactions, between each small-molecule metabolite and the receptor’s active site. The binding affinity of the metabolites to the target protein complex was assessed by comparing binding energies.

### Data analysis

GraphPad Prism software was used to statistically analyze all of the study’s data. Experimental outcomes are denoted as mean ± SD. When data were normally distributed and had equal variances, the unpaired Student’s *t* test was employed for comparisons between two groups. One-way ANOVA was performed for comparisons among several groups, followed by Tukey’s post hoc test to evaluate significant differences. A *P*-value < 0.05 was considered statistically significant.

## Supplementary information

Supplementary information

## Data Availability

The datasets generated and/or analyzed during the current study are not publicly available due to institutional data management policies and ongoing related research work, but are available from the corresponding author upon reasonable request.
