# Supplementary material for: Sarcandra glabra alleviates intestinal injury via modulating gut microbiota and suppressing the IL-6/SOCS3 signaling pathway
Source: NPJ Sci Food. 2026 Jul 1;10:325. doi: 10.1038/s41538-026-00968-4 (PMC13691109; doi:10.1038/s41538-026-00968-4)
Supplement: Supplementary file 1 [file 41538_2026_968_MOESM1_ESM.pdf]

Table S1 Primer sequences

Primer name	Sequences (5' to 3')
GAPDH-Forward	TGCACCACCAACTGCTTAGC
GAPDH-Reverse	GGCATGGACTGTGGTCATGAG
ZO1-Forward	GTGCTGGCTTGGTCTGTTTG
ZO1-Reverse	TCTCCCACTCTGTCTCCAGG
OCLN-Forward	CACCACACCTACACTCCCG
OCLN-Reverse	AAACCAATCTGCTGCGTCCT
CLDN1-Forward	GGTCAGGCTCTCTTCACTGG
CLDN1-Reverse	GGCCTTGGTGTTGGGTAAGA

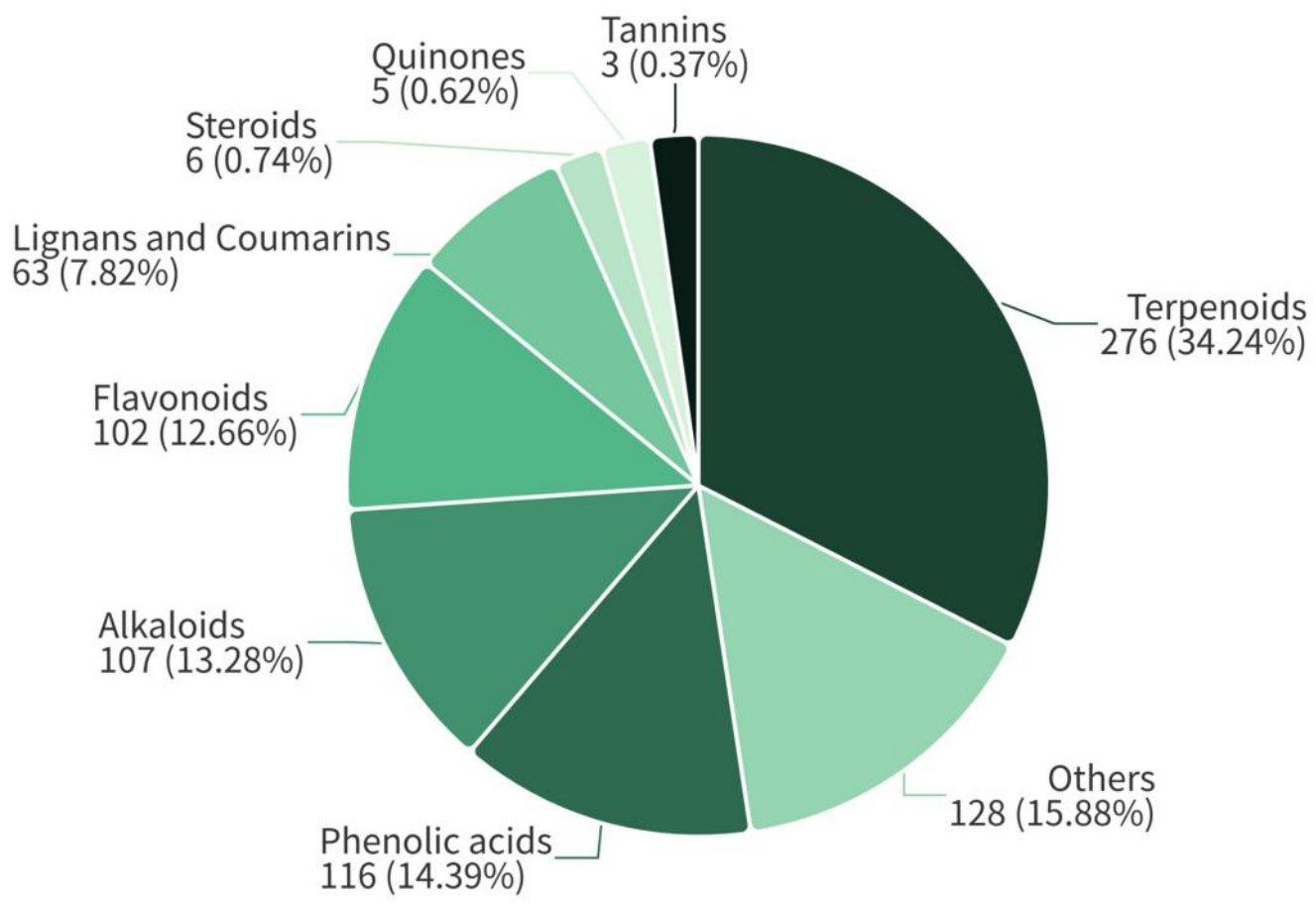

Figure S1. The proportion of various chemical components in Sg.

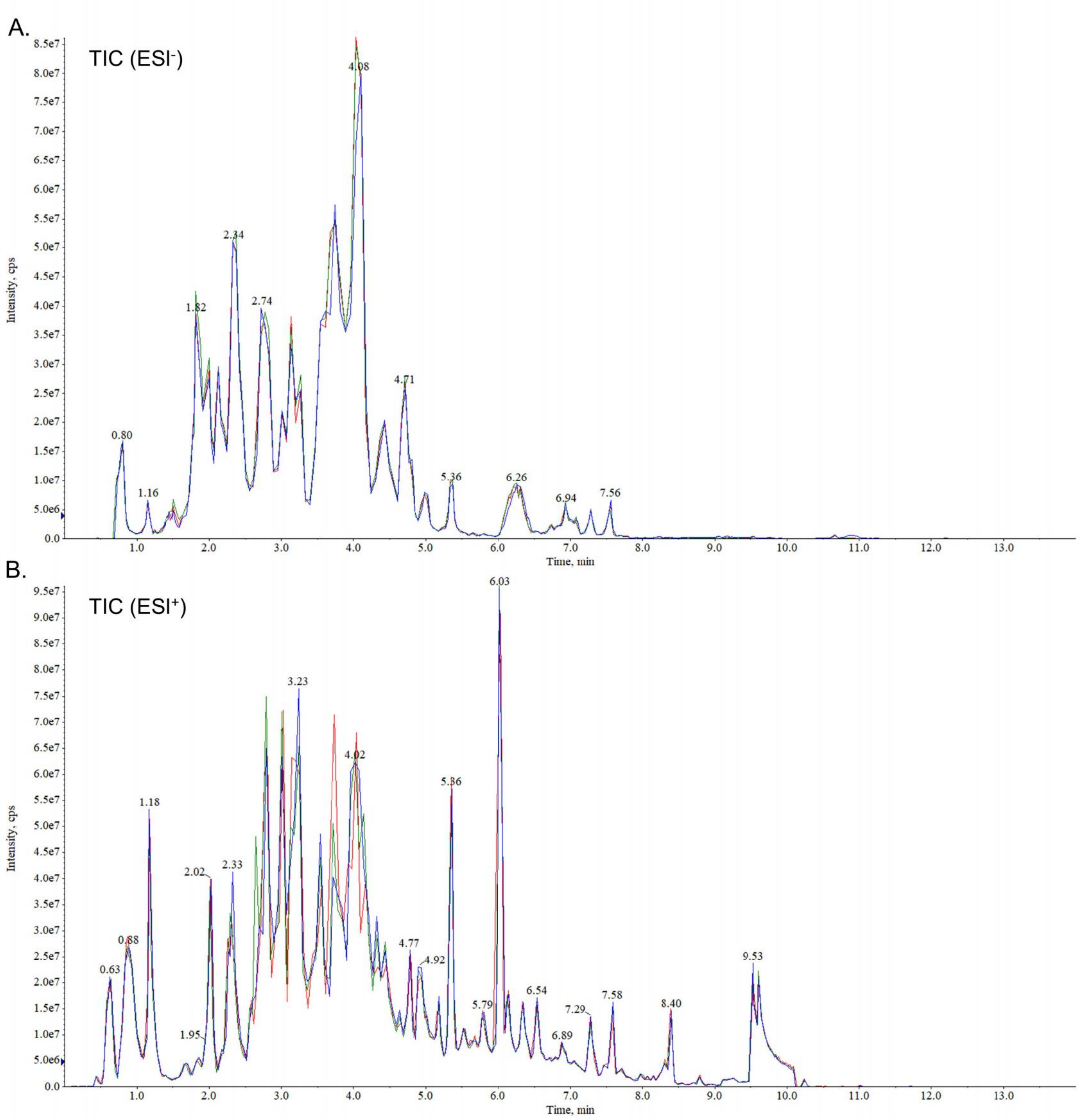

Figure S2. The total ion current chromatograms of 3 batches of Sg. A. Negative ion mode; B. Positive ion mode.

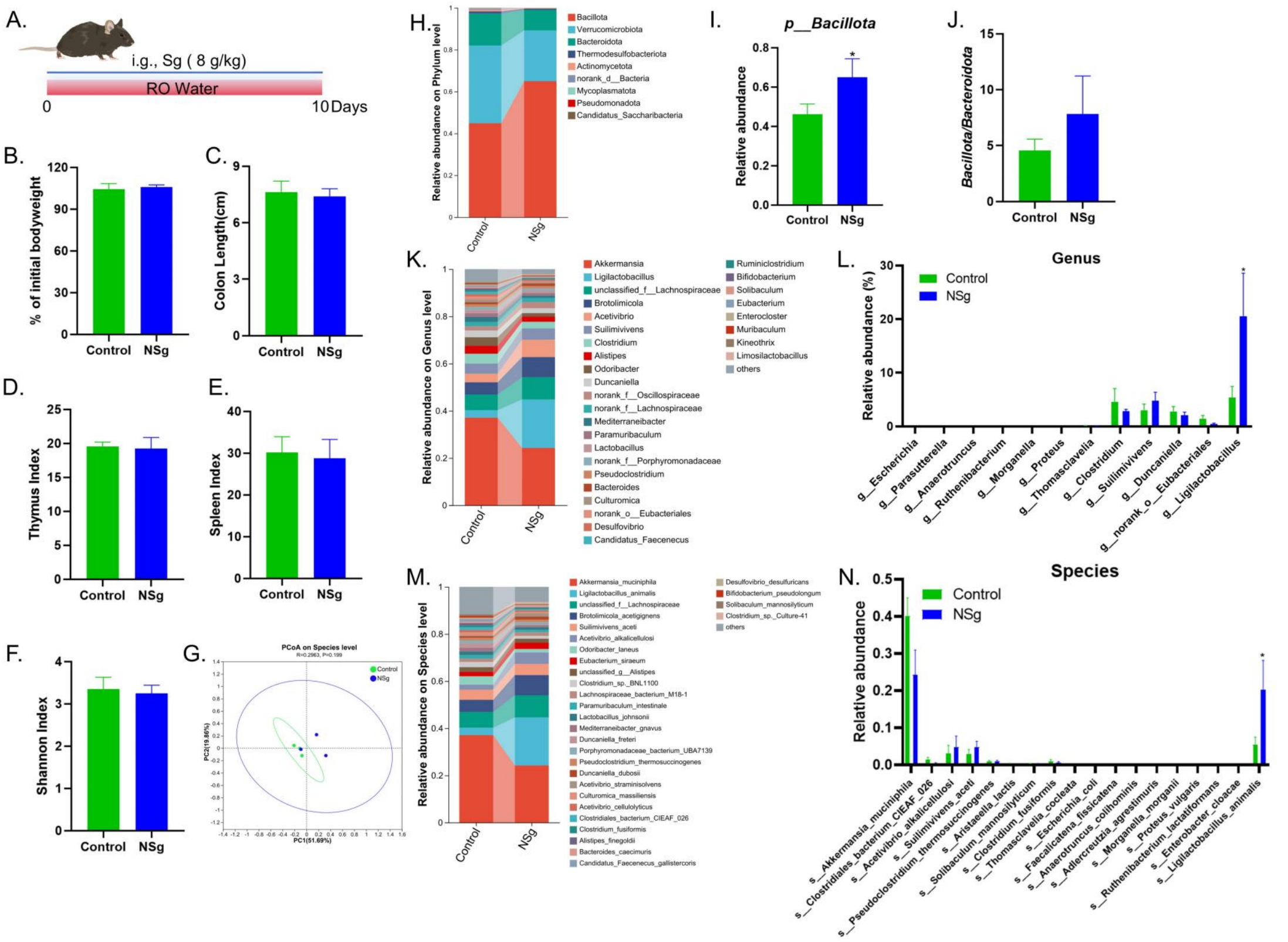

Figure S3. The influence of Sg on the gut microbiota of normal mice. A. The method of Sg intervention in normal mice; B. Changes in the body weight; C. Changes in the colon length; D. Changes in the thymus index; E. Changes in the spleen index; F. Changes in the Shannon index; G. PCoA analysis in different groups; H. Bar plot of community at phylum level; I. The relative abundance of Bacillota; J. The ratio of Bacillota/Bacteroidota in different groups; K. Bar plot of community at genus level; L. The relative abundances of 12 bacterial genera in the two groups; M. Bar plot of community at species level; N. The relative abundances of 18 bacterial strains in the two groups. n=3. *vs*. Control, *$P < 0.05$.

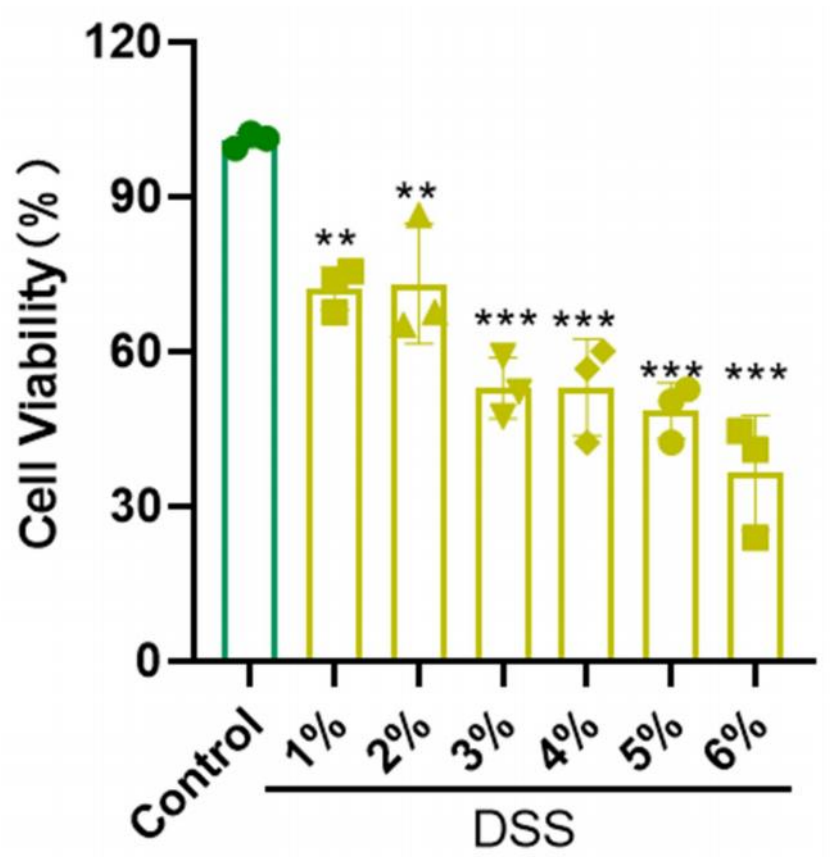

Figure S4. Effects of different concentrations of DSS on the viability of NCM460 cells. n=3. *vs*. Control, *$P<0.05$.

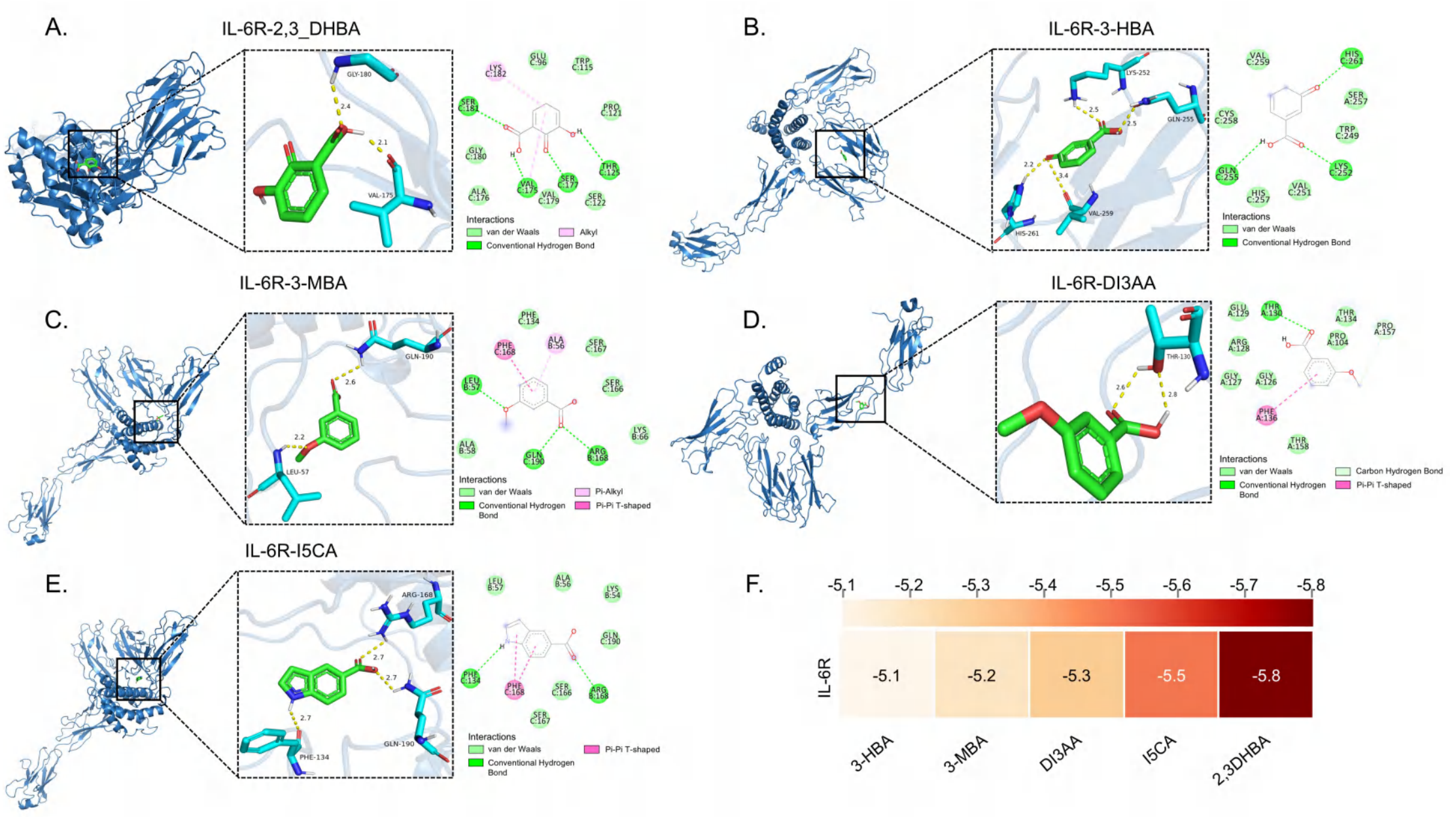

Figure S5. The binding situations of five key metabolites with the IL-6/IL-6 alpha receptor/gp130 complex. A. Molecular docking of 2,3-DHBA with protein complex; B. Molecular docking of 3-HBA with protein complex; C. Molecular docking of 3-MBA with protein complex; D. Molecular docking of DI3AA with protein complex; E. Molecular docking of I5CA with protein complex; F. The heat map of docking scores of (A-F).

Full unedited gel for Figure 4J

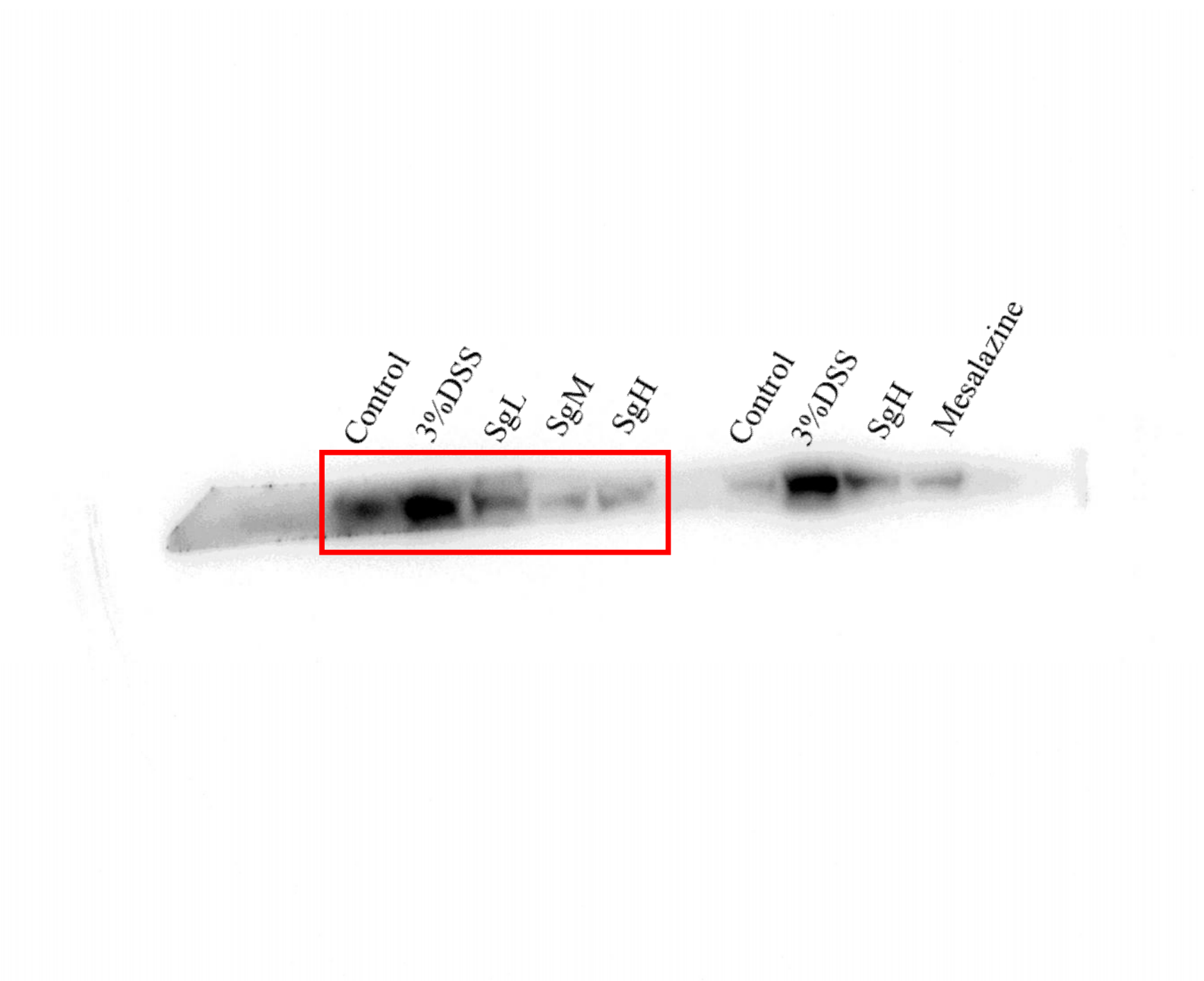

1.p-STAT3-88 kDa (chemi)

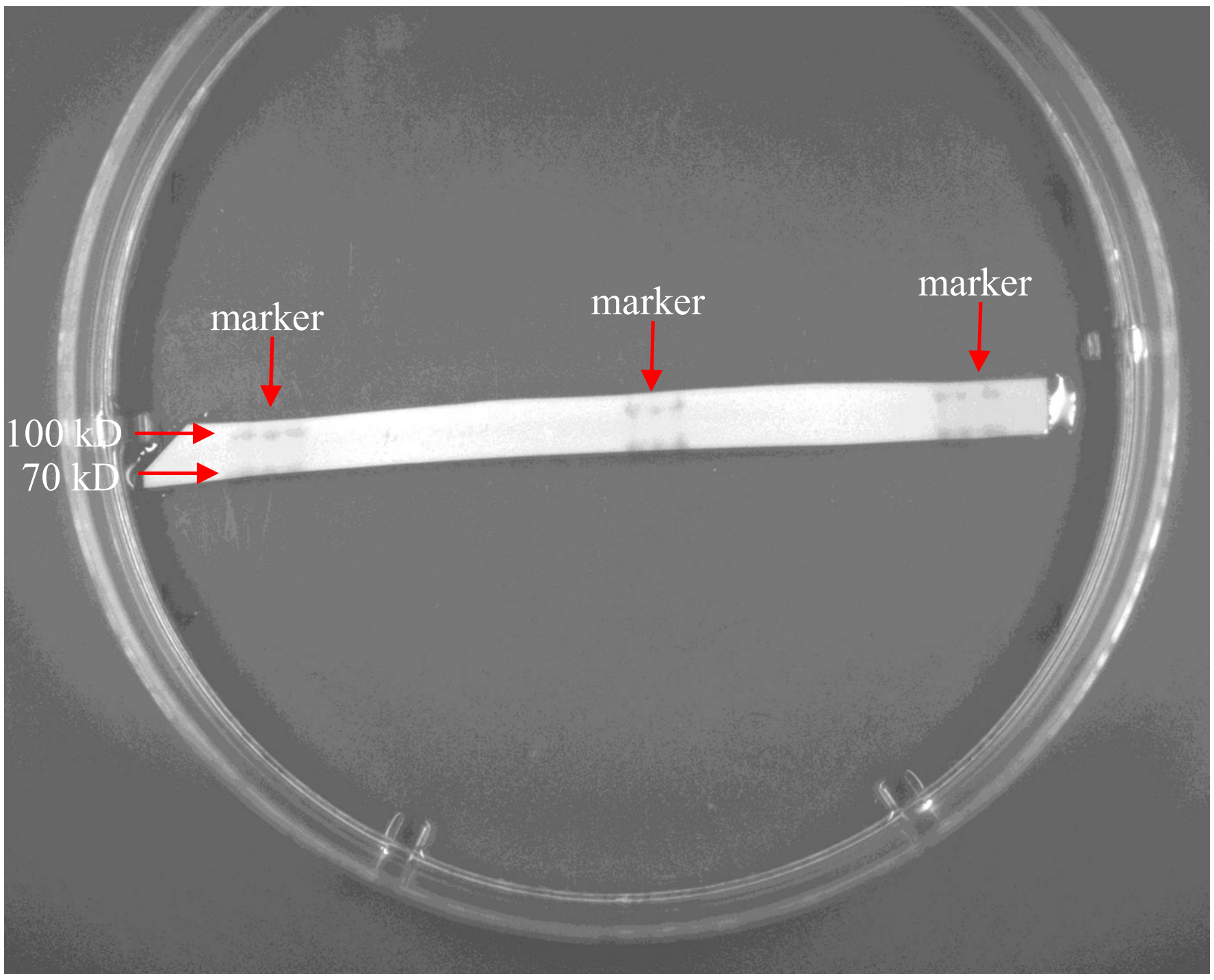

1.p-STAT3-88 kDa (white)

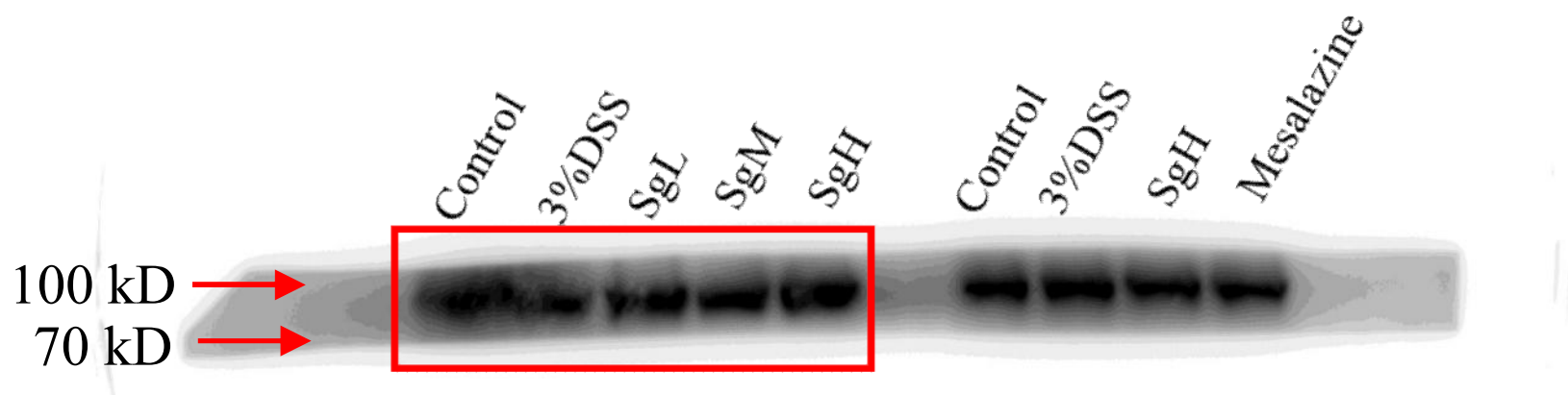

2.STAT3-88 kDa

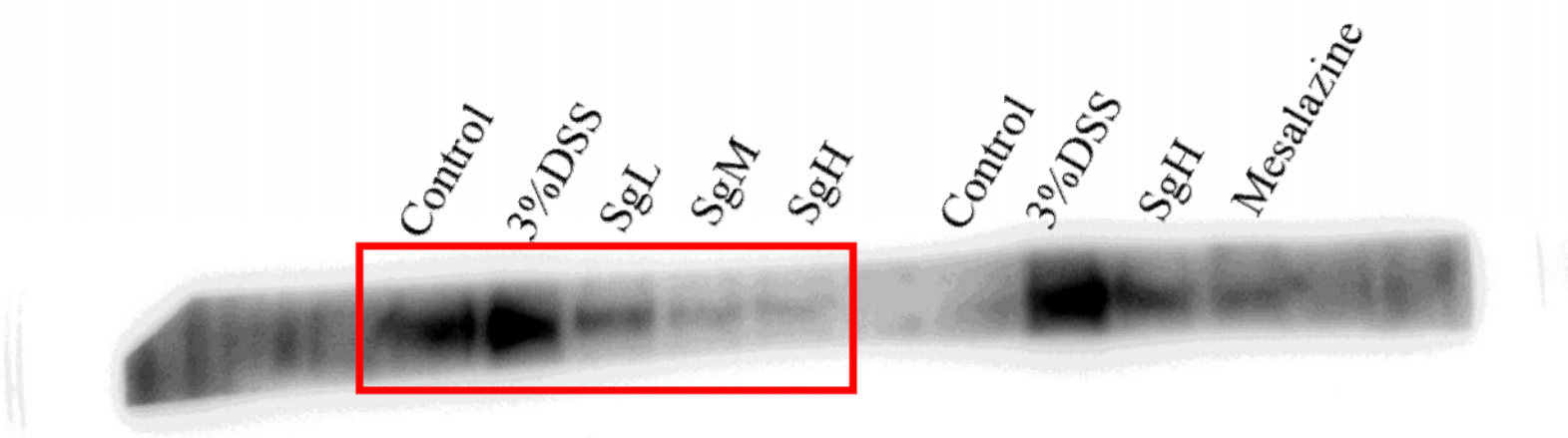

3.p-JAK2-120 kDa (chemi)

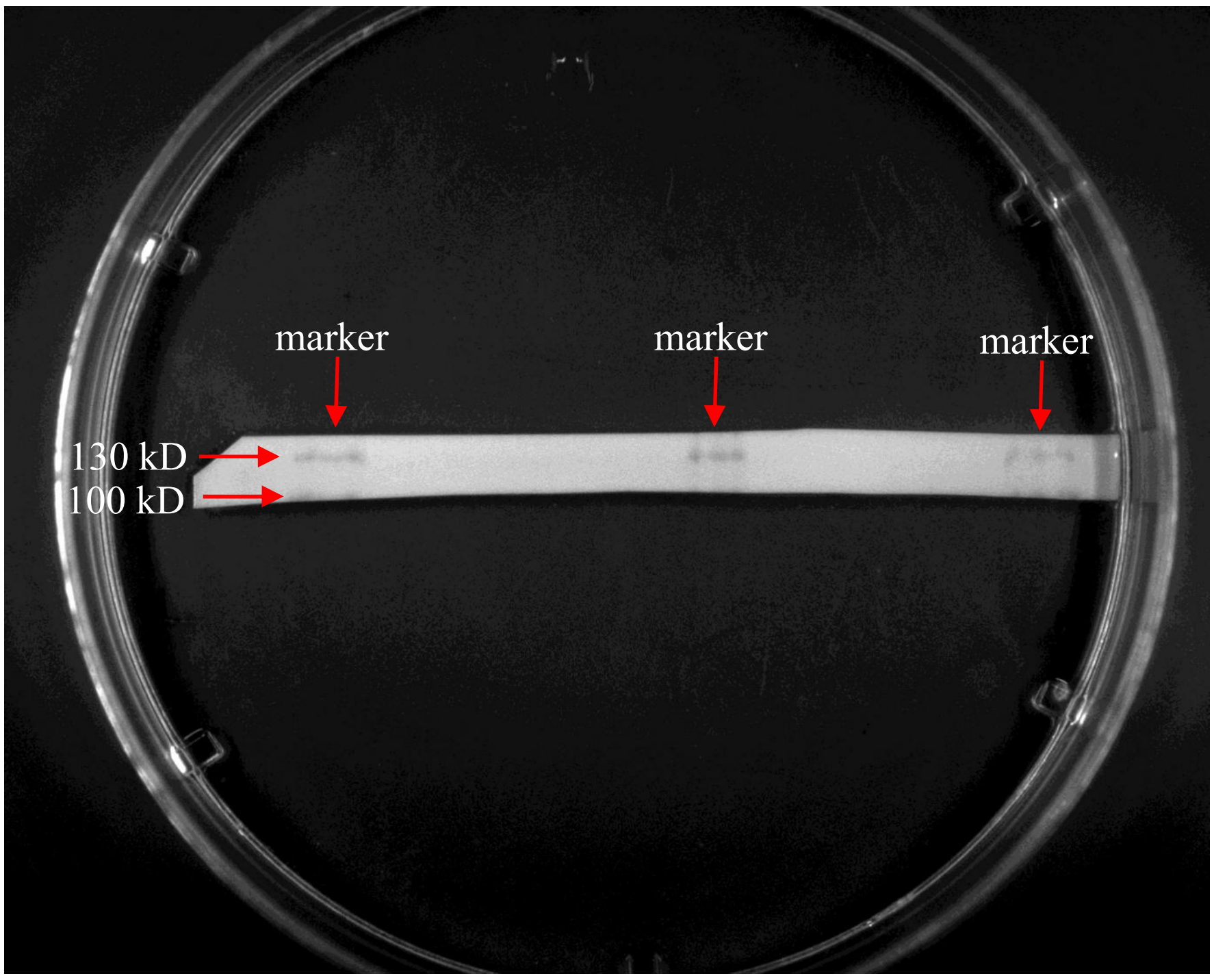

3.p-JAK2-120 kDa (white)

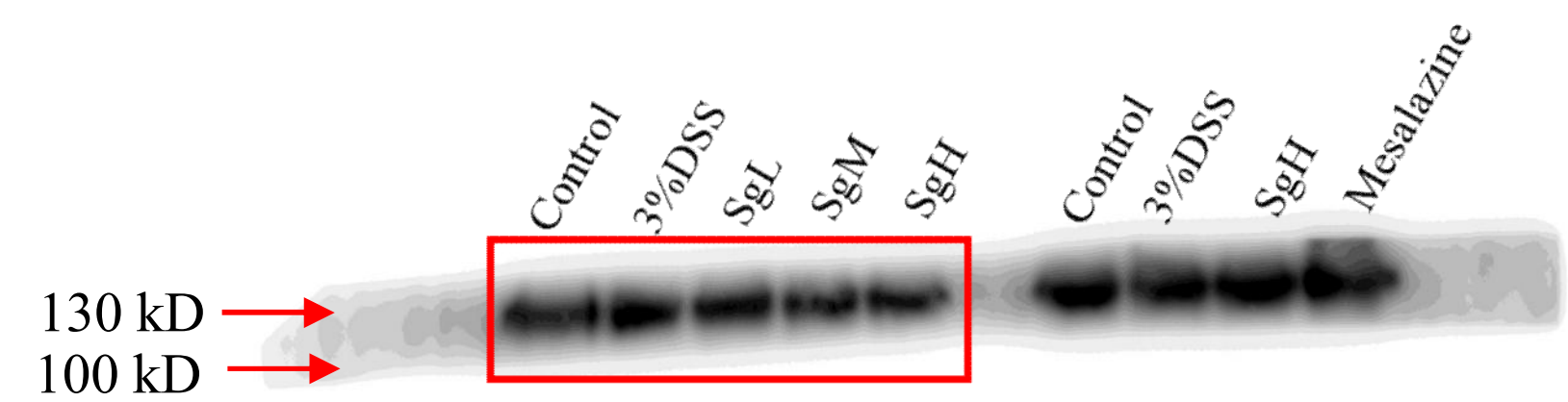

4.JAK2-120 kDa

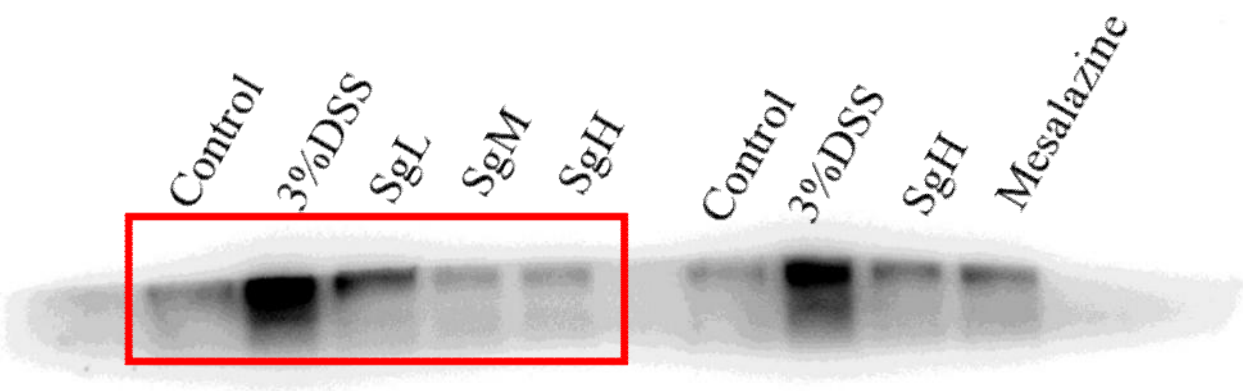

5.IL6R-78 kDa (chemi)

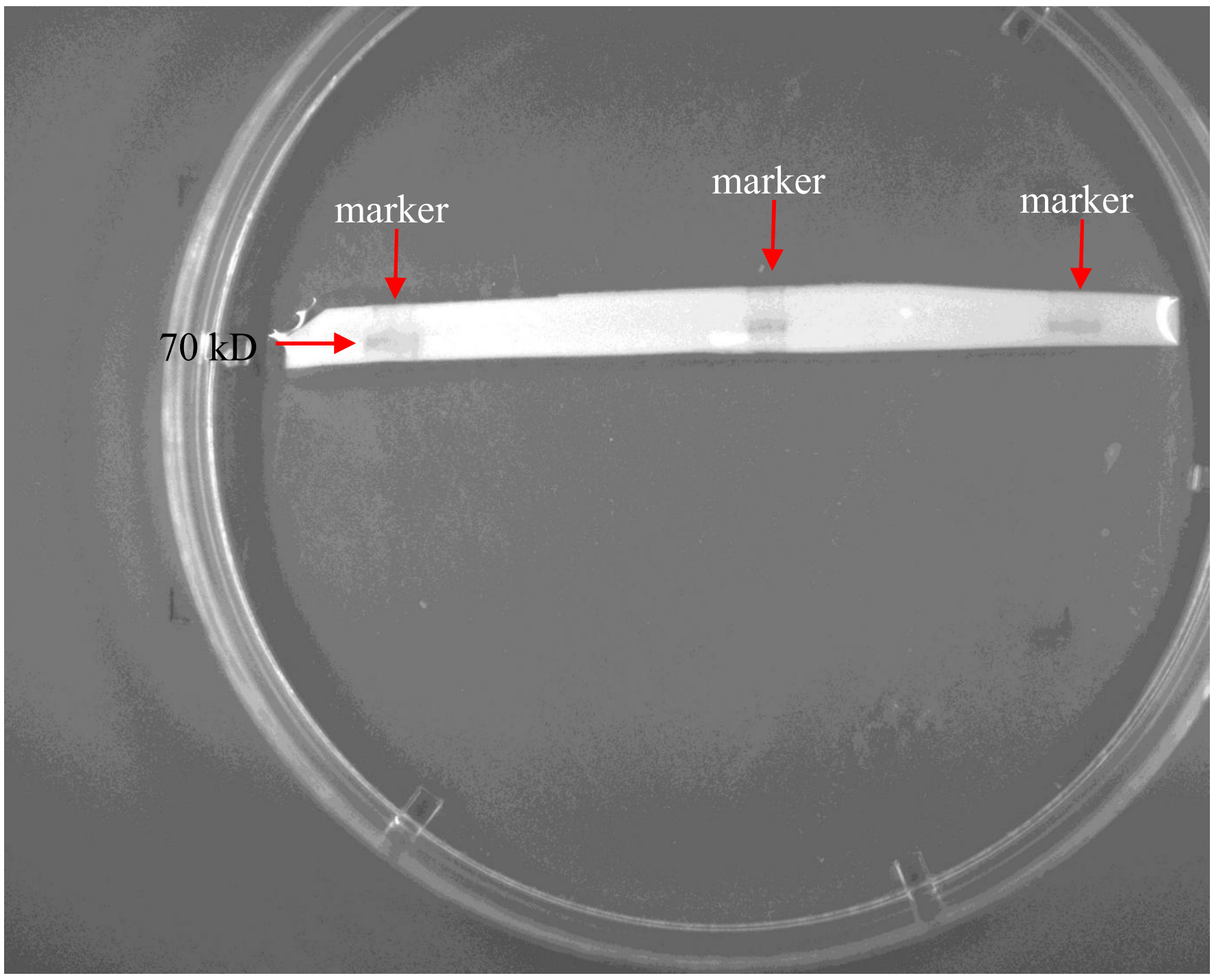

5.IL6R-78 kDa (white)

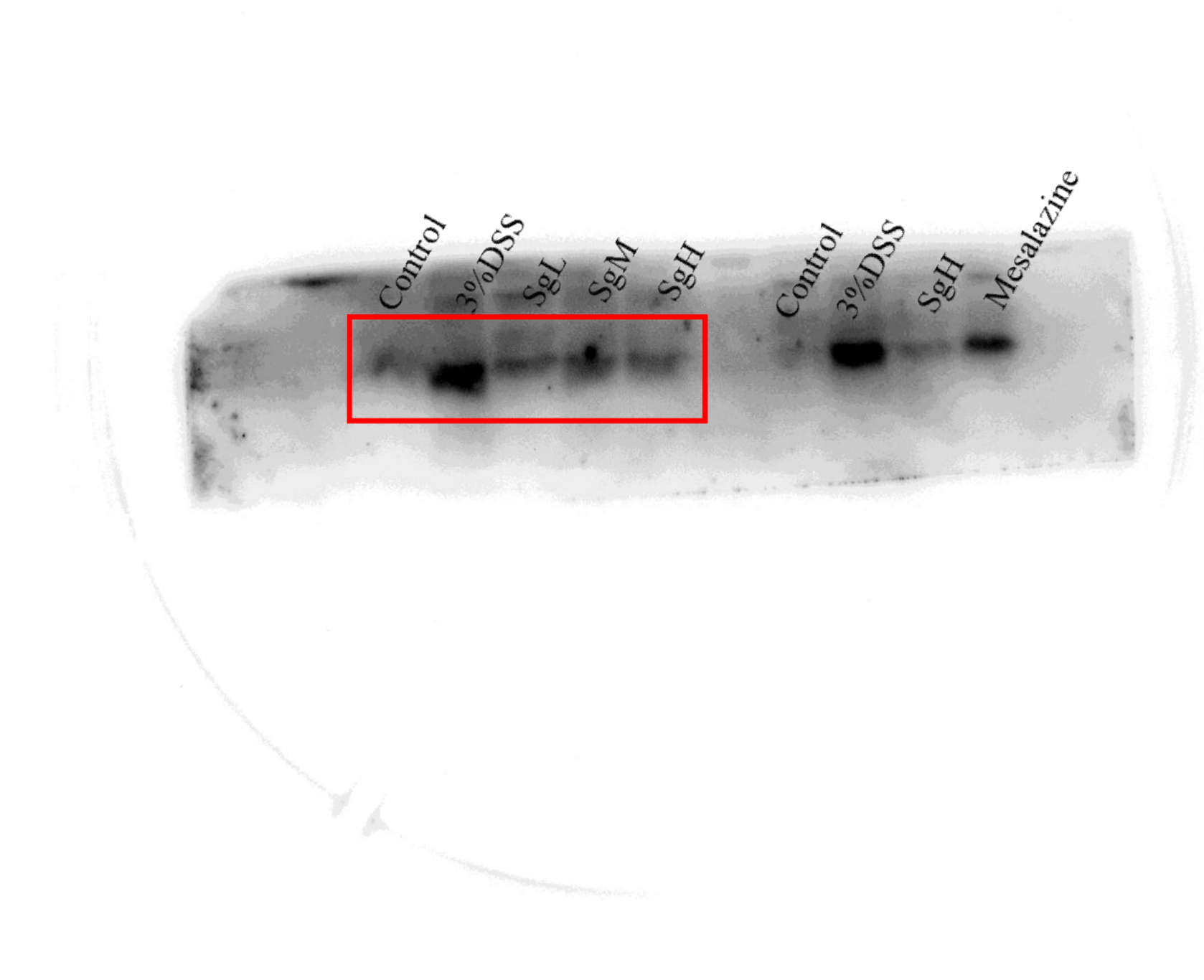

6.SOCS3-25 kDa (chemi)

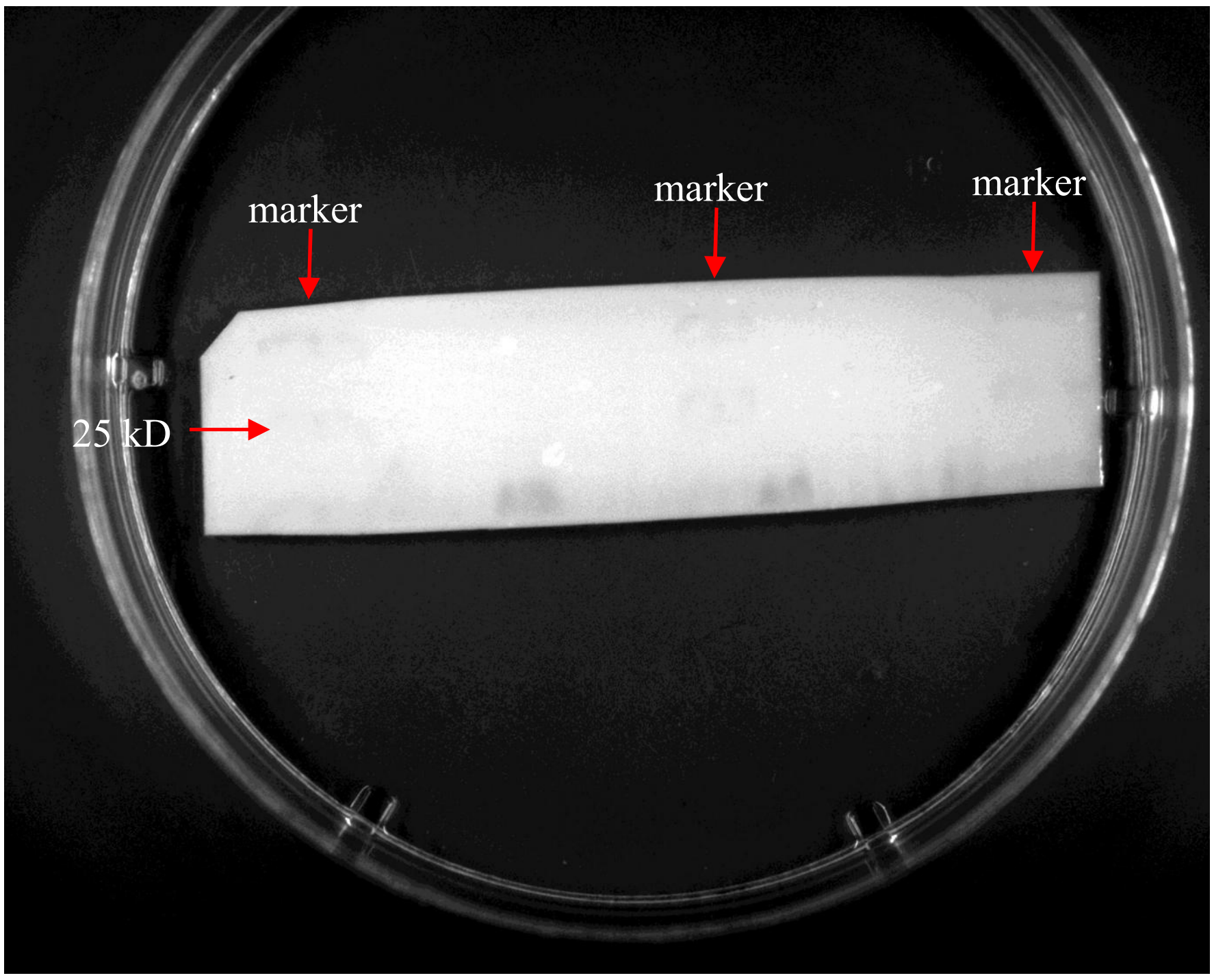

6.SOCS3-25 kDa (white)

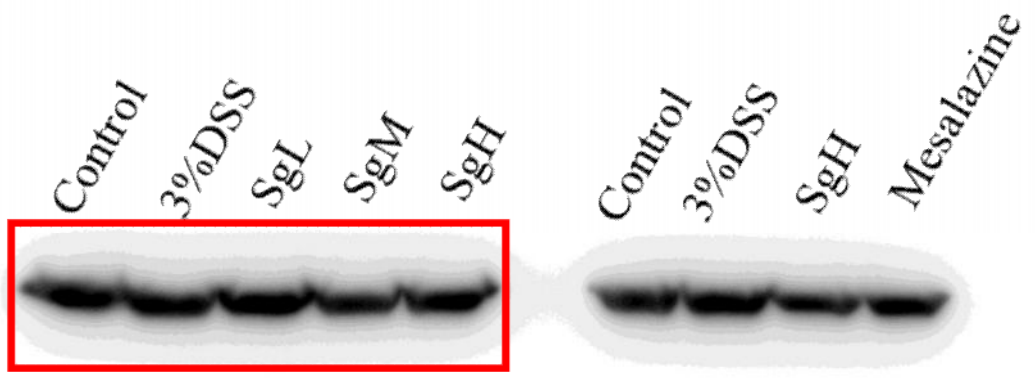

7.β-actin-43 kDa (chemi)

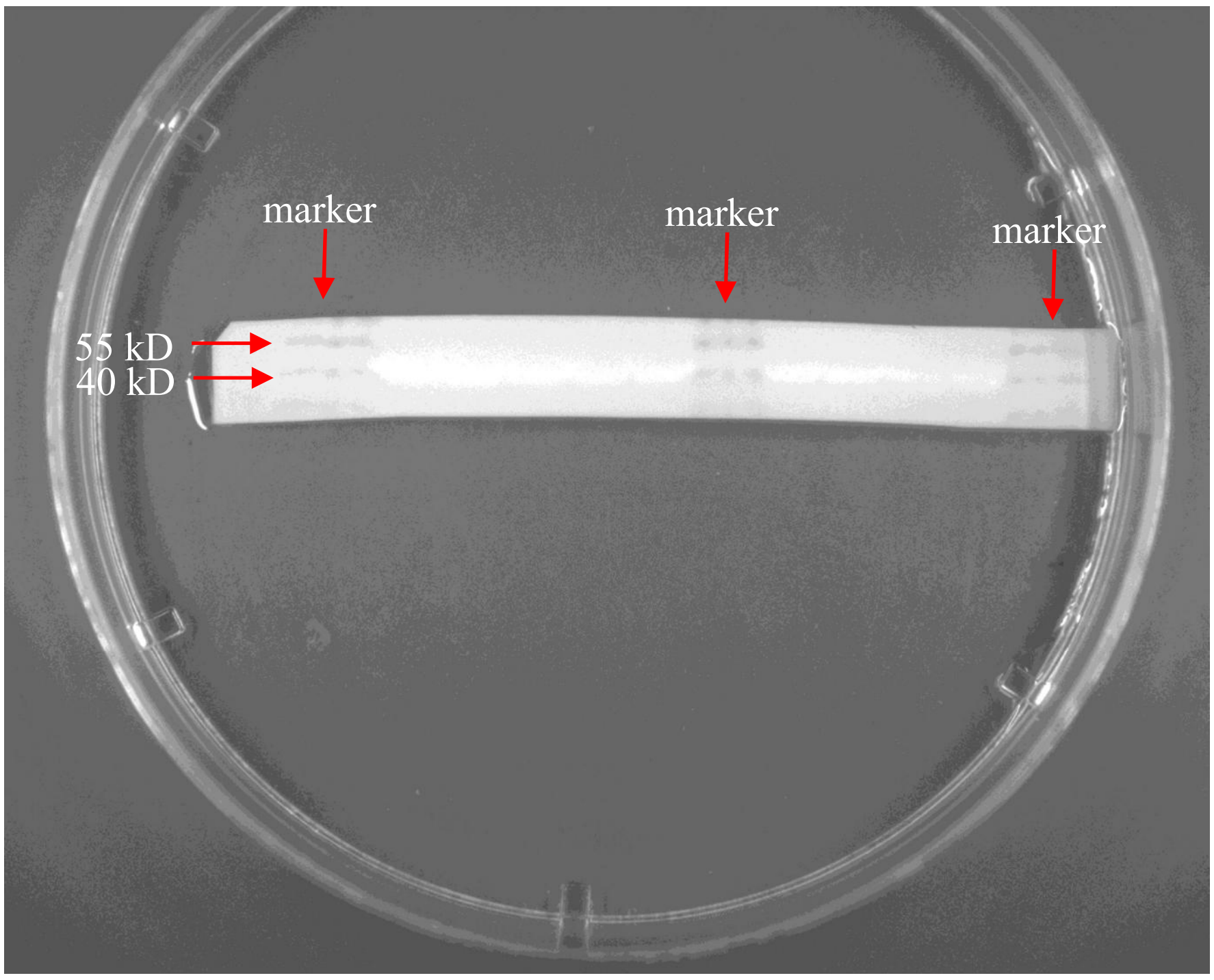

7. β-actin-43 kDa (white)

Full unedited gel for Figure 8H

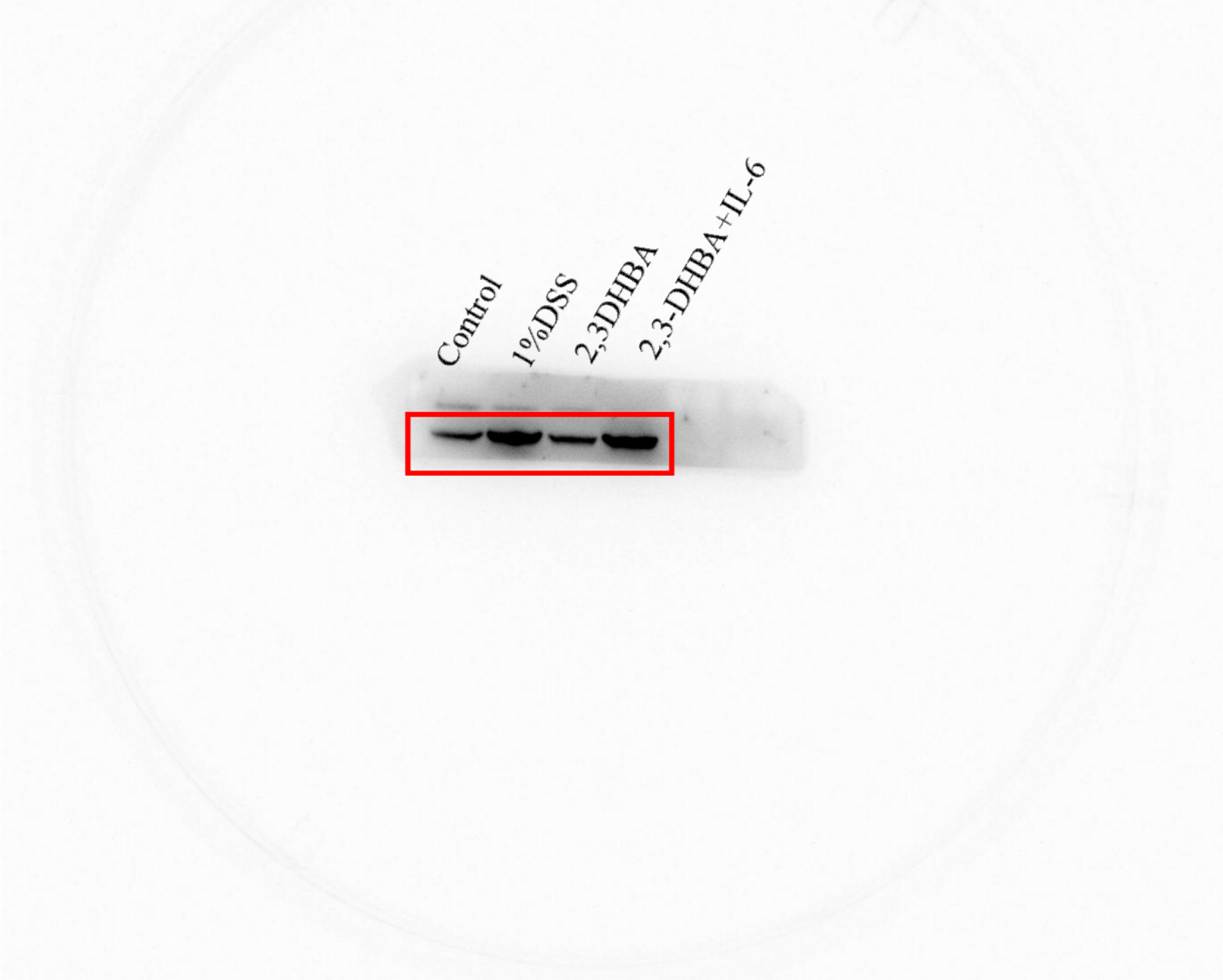

1.p-STAT3-88 kDa (chemi)

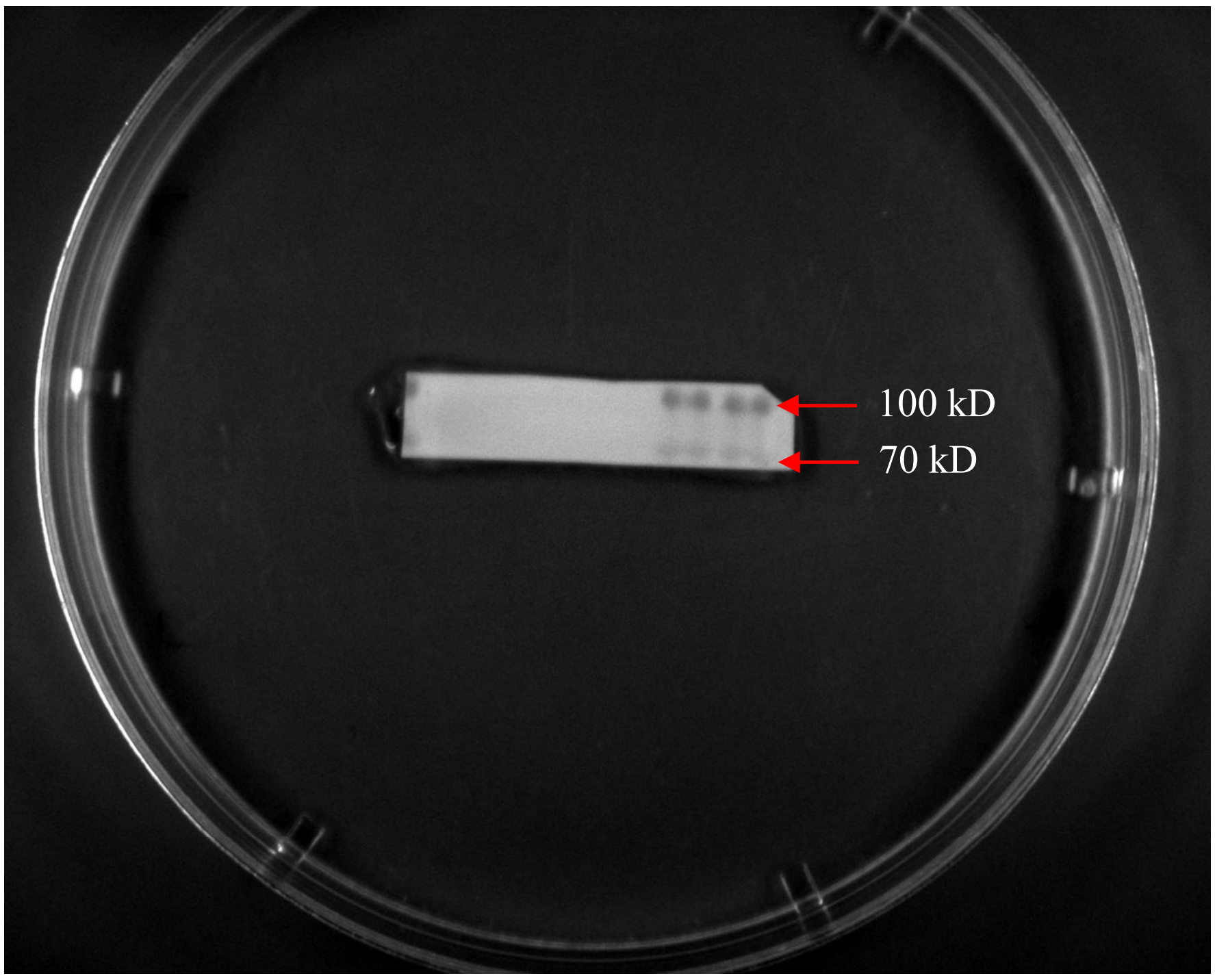

1.p-STAT3-88 kDa (white)

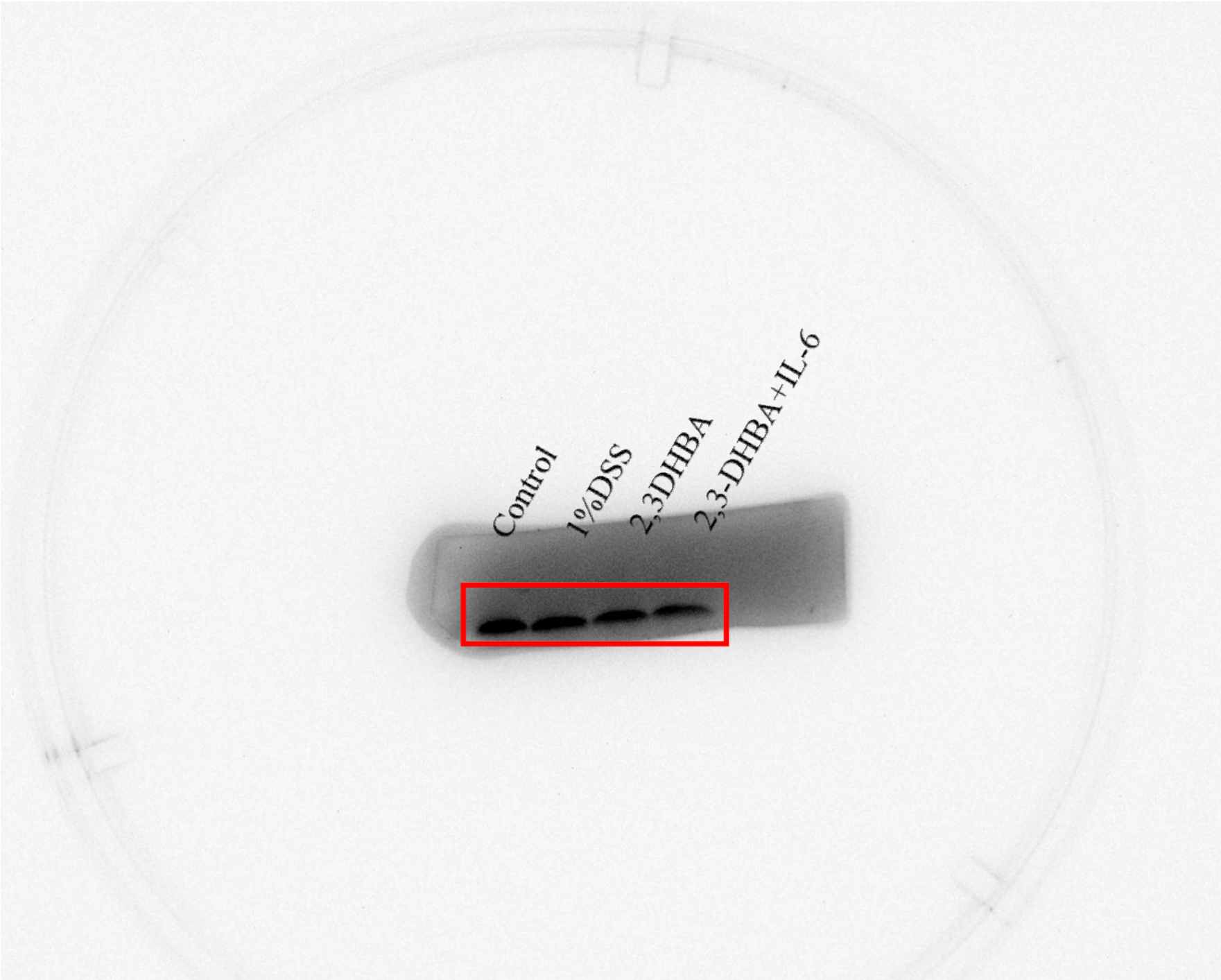

2.STAT3-88 kDa (chemi)

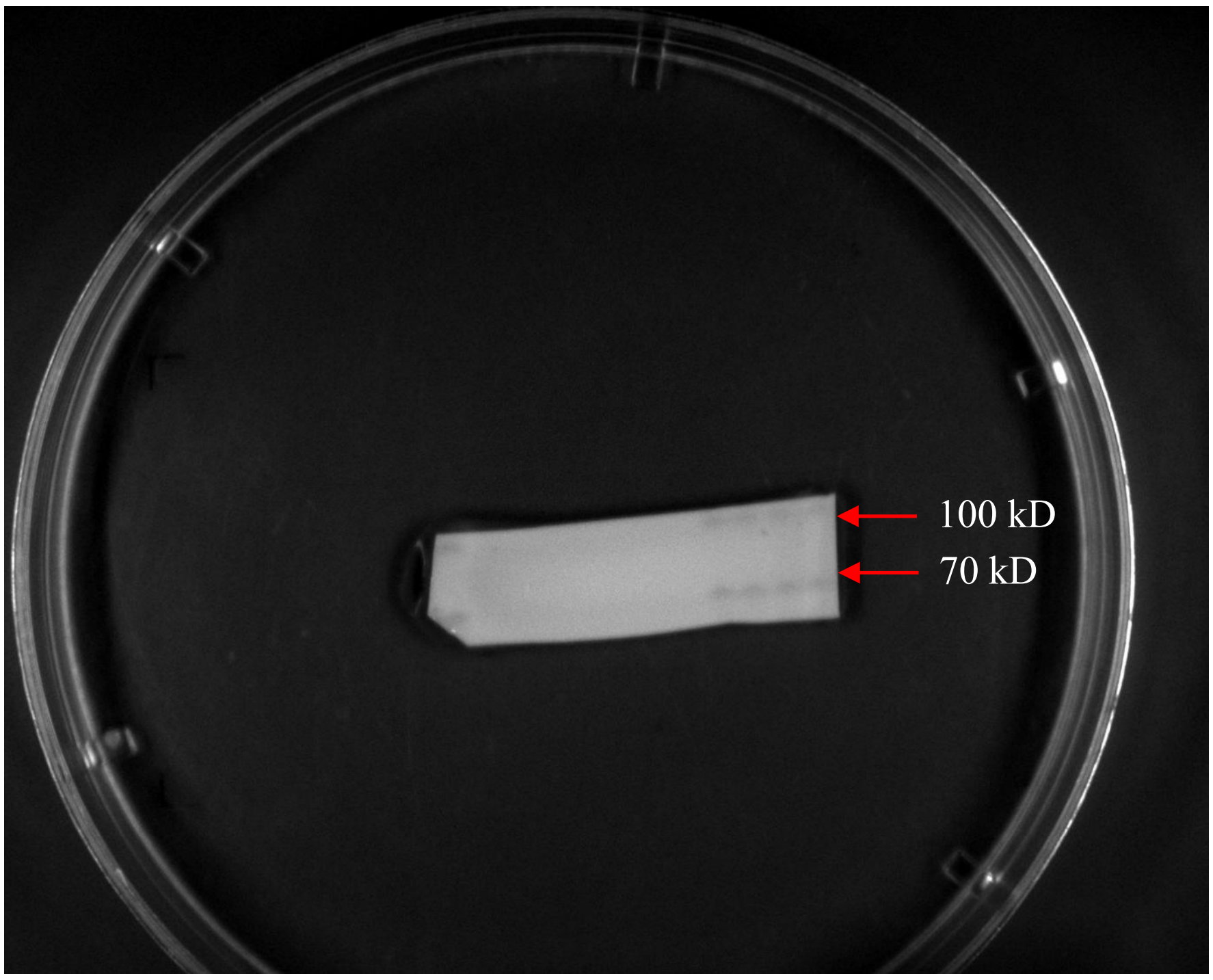

2.STAT3-88 kDa (white)

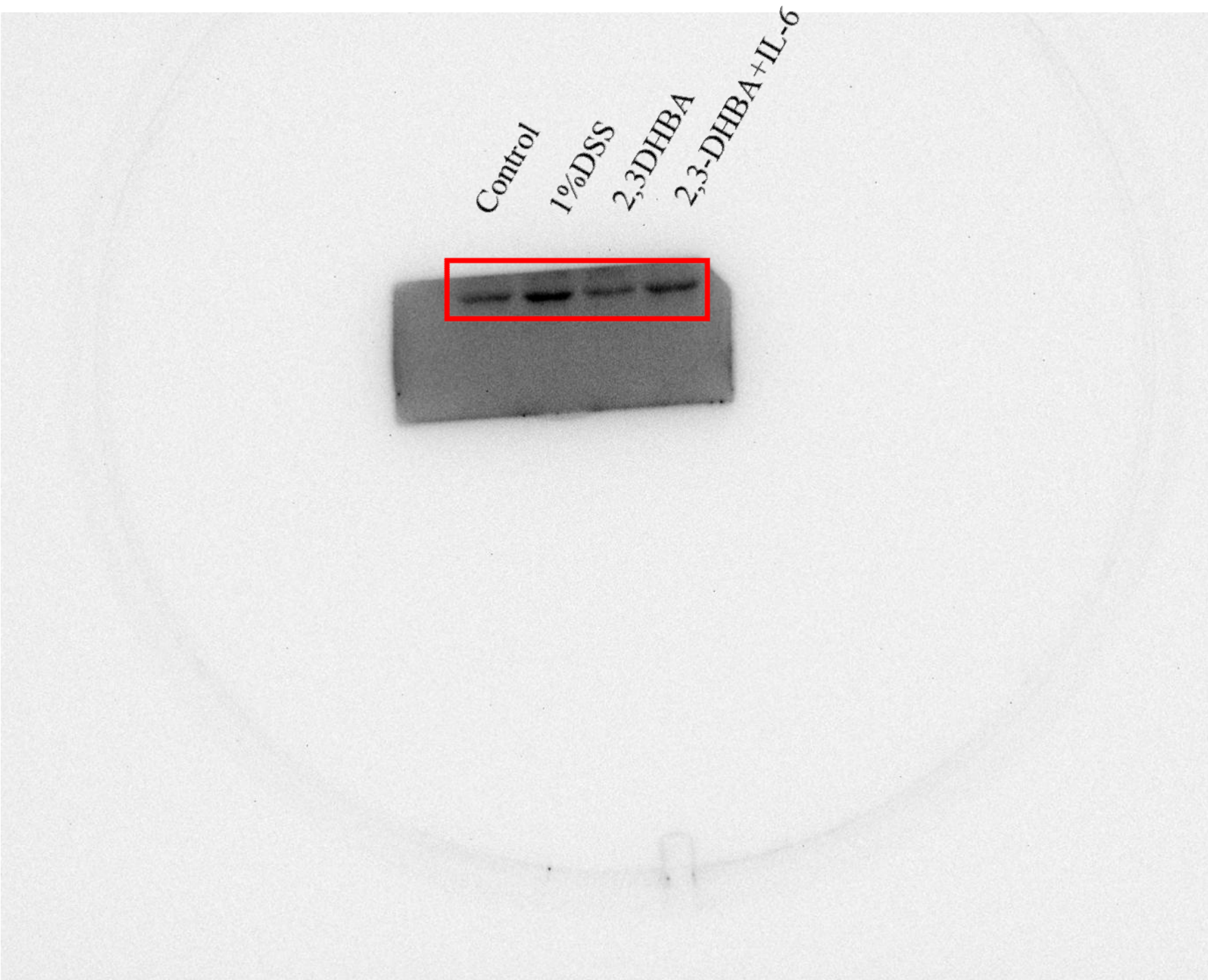

3.p-JAK2-120 kDa (chemi)

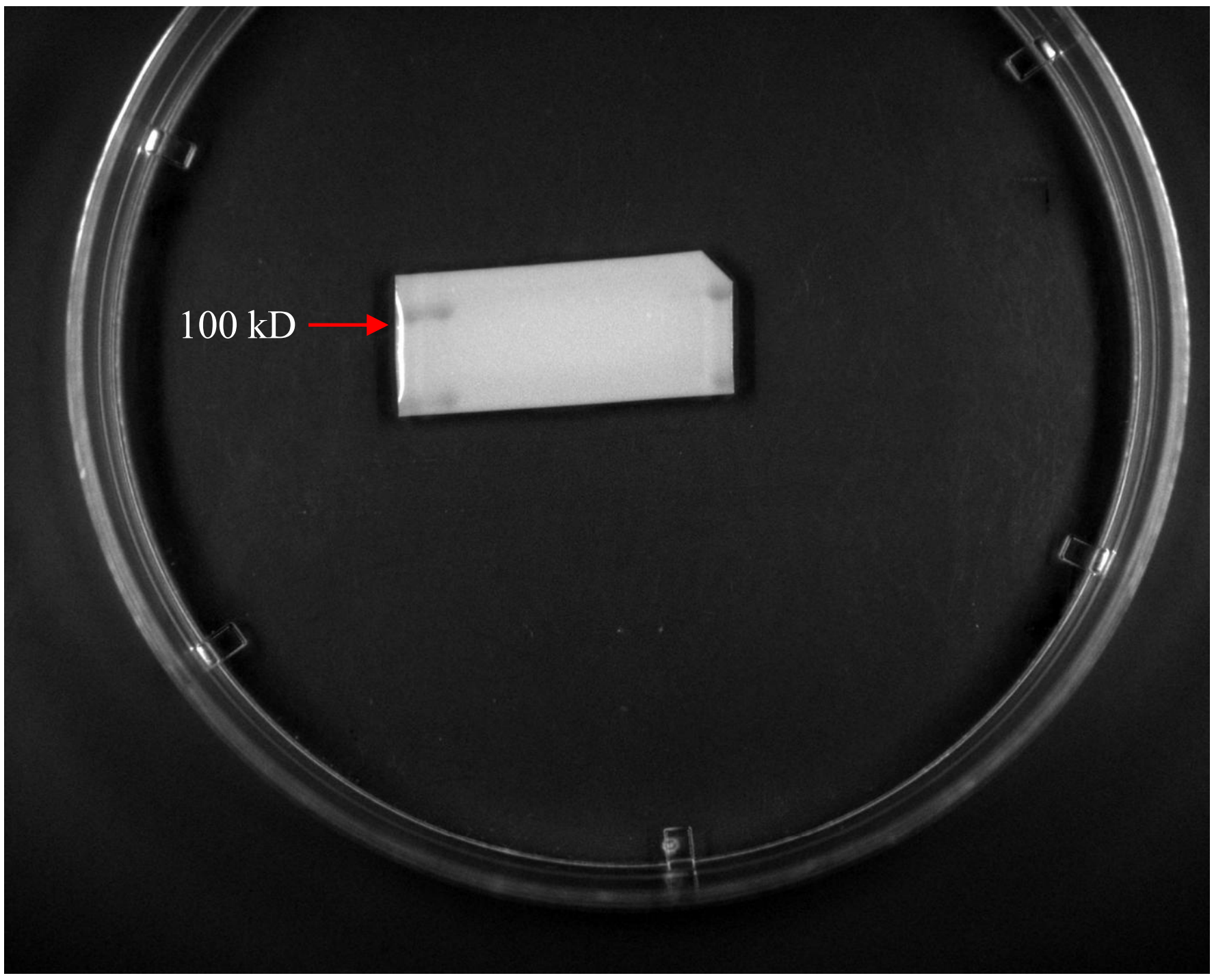

3.p-JAK2-120 kDa (white)

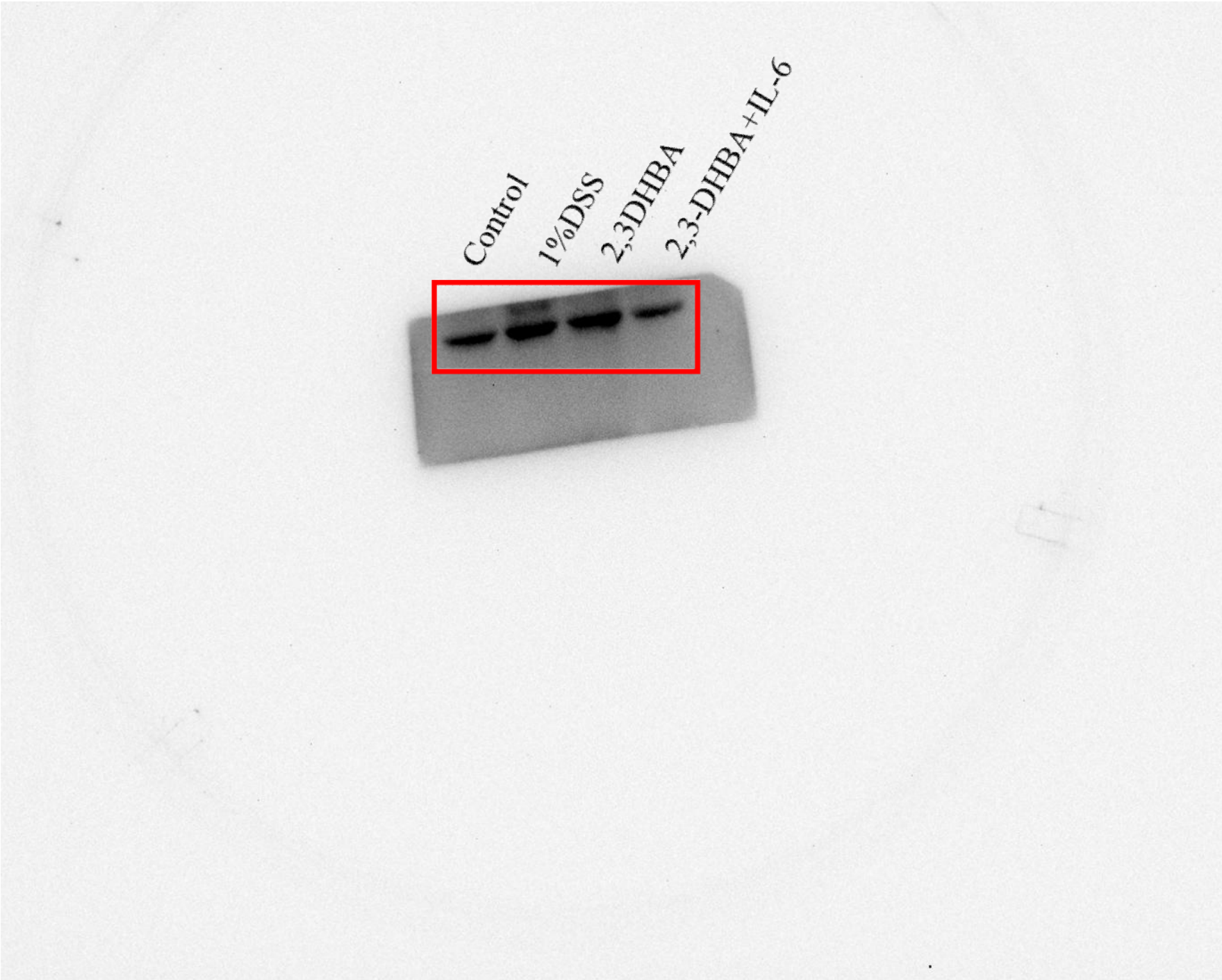

4.JAK2-120 kDa (chemi)

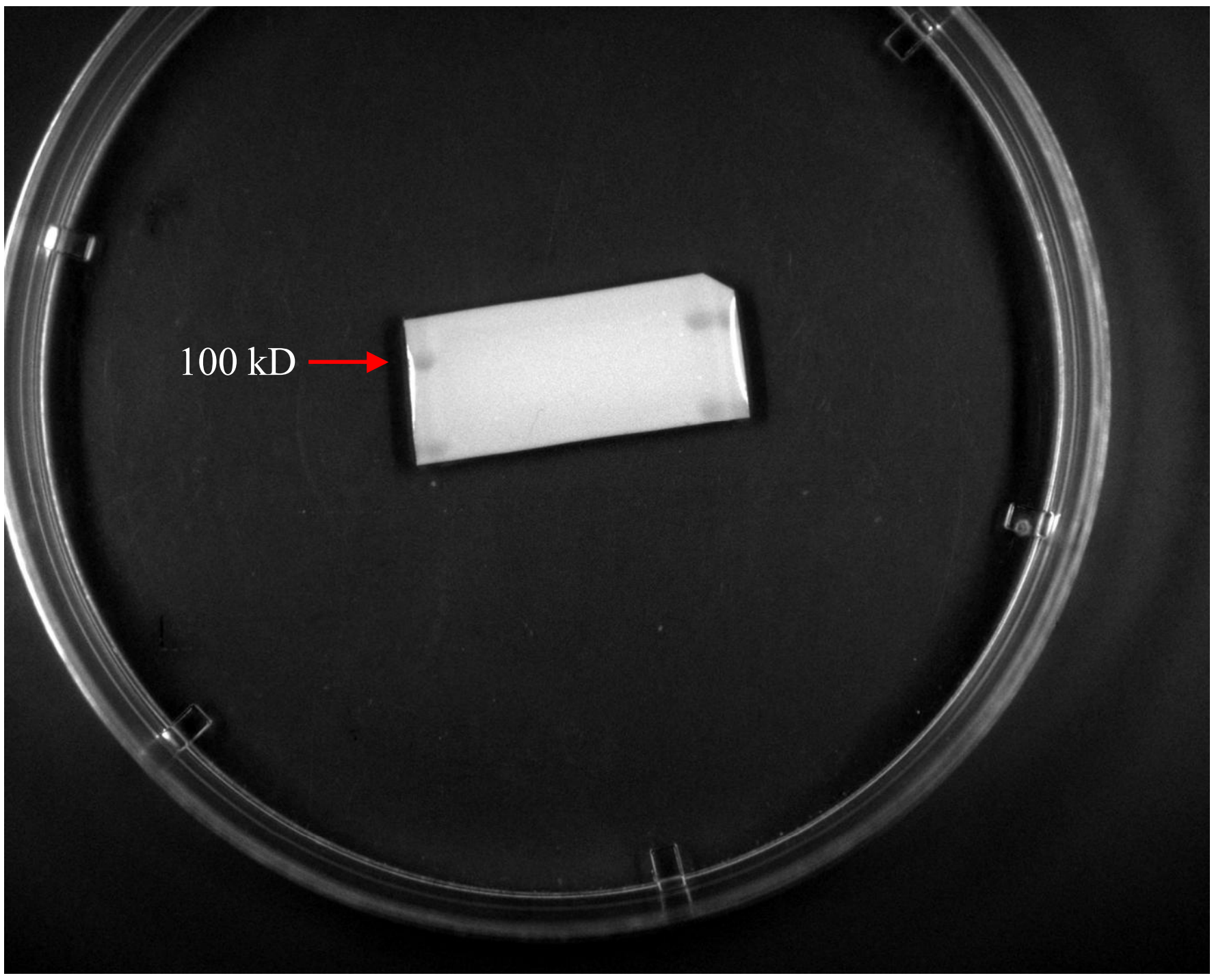

4.JAK2-120 kDa (white)

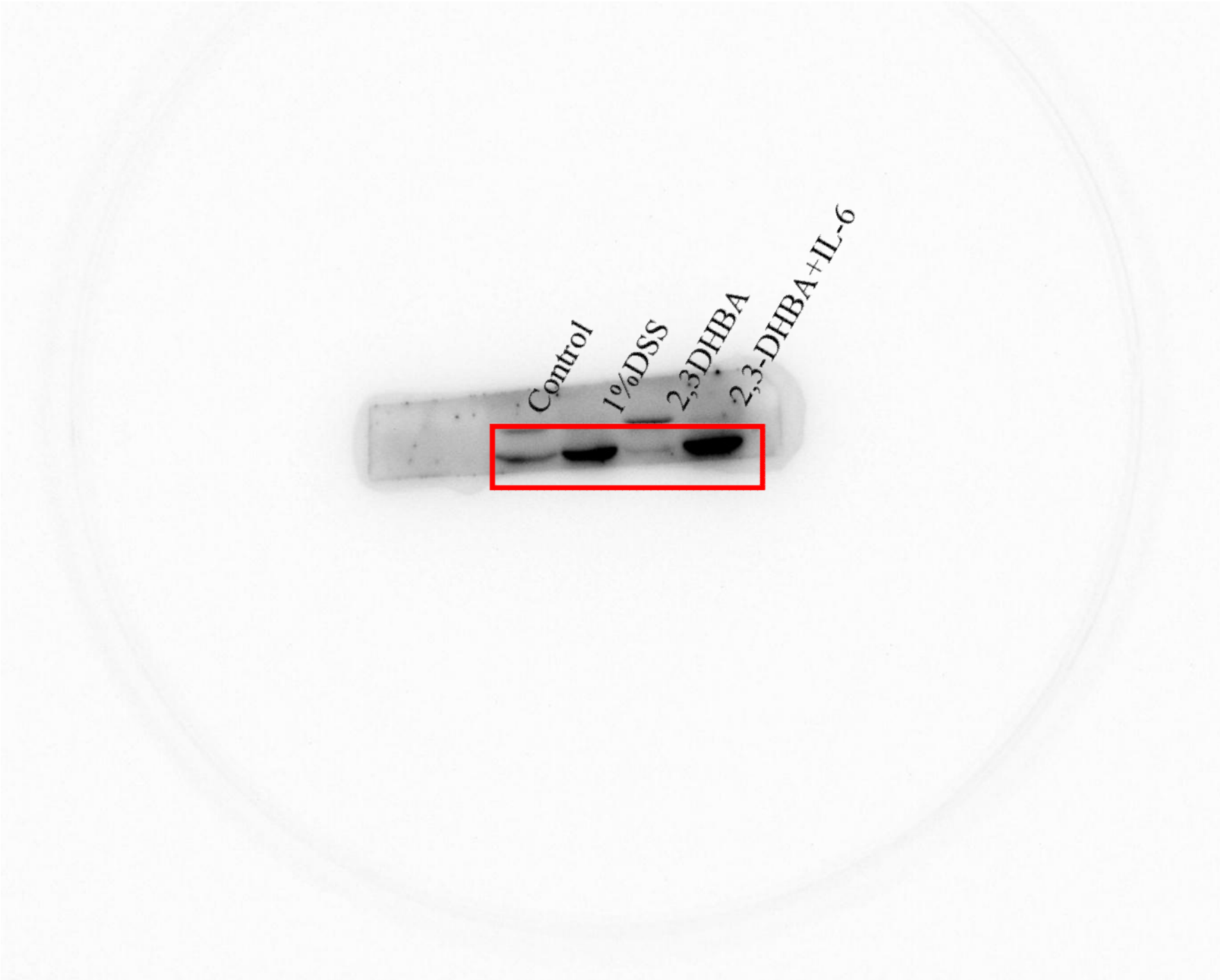

5.IL6R-78 kDa (chemi)

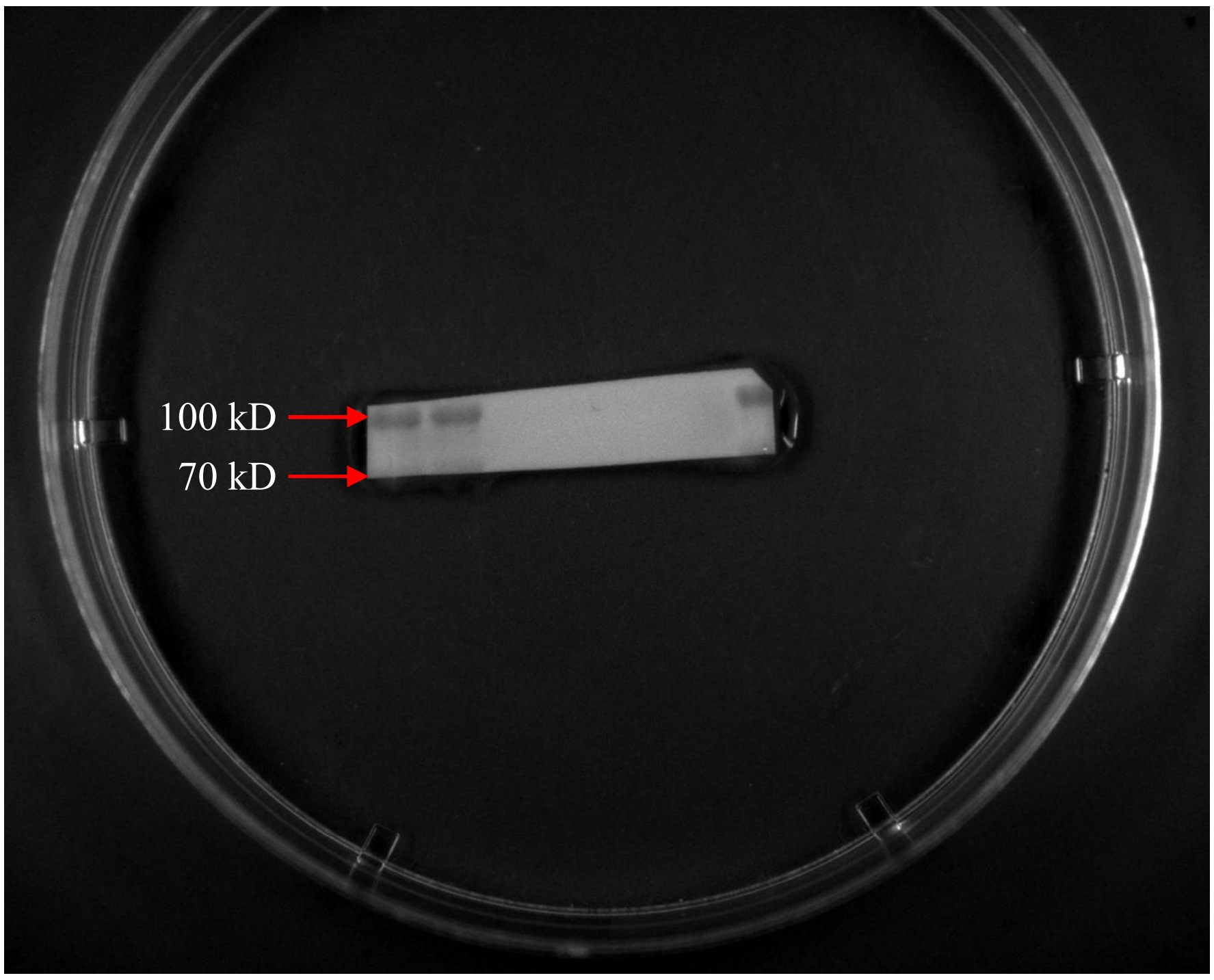

5. IL6R-78 kDa (white)

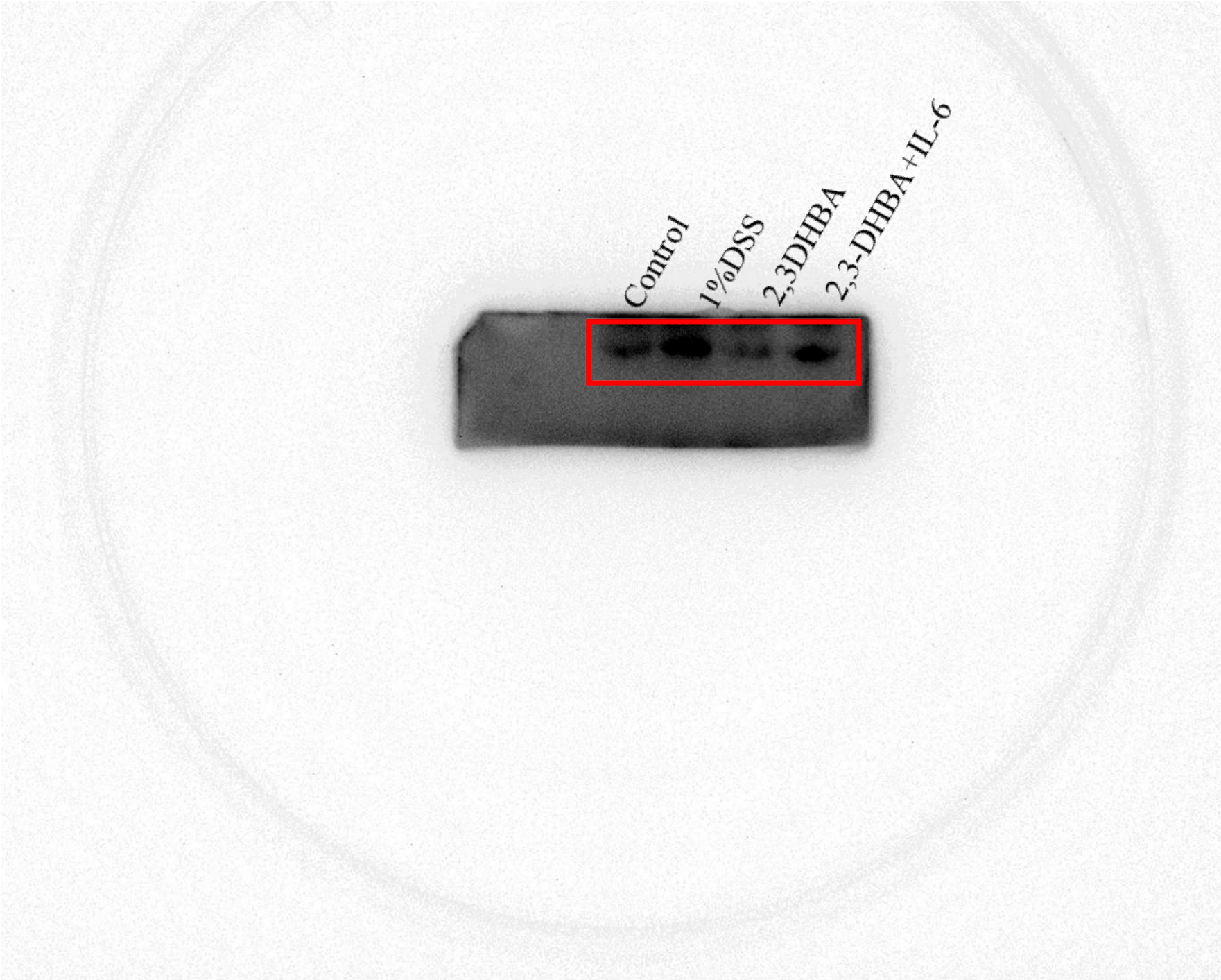

6. SOCS3-25 kDa (chemi)

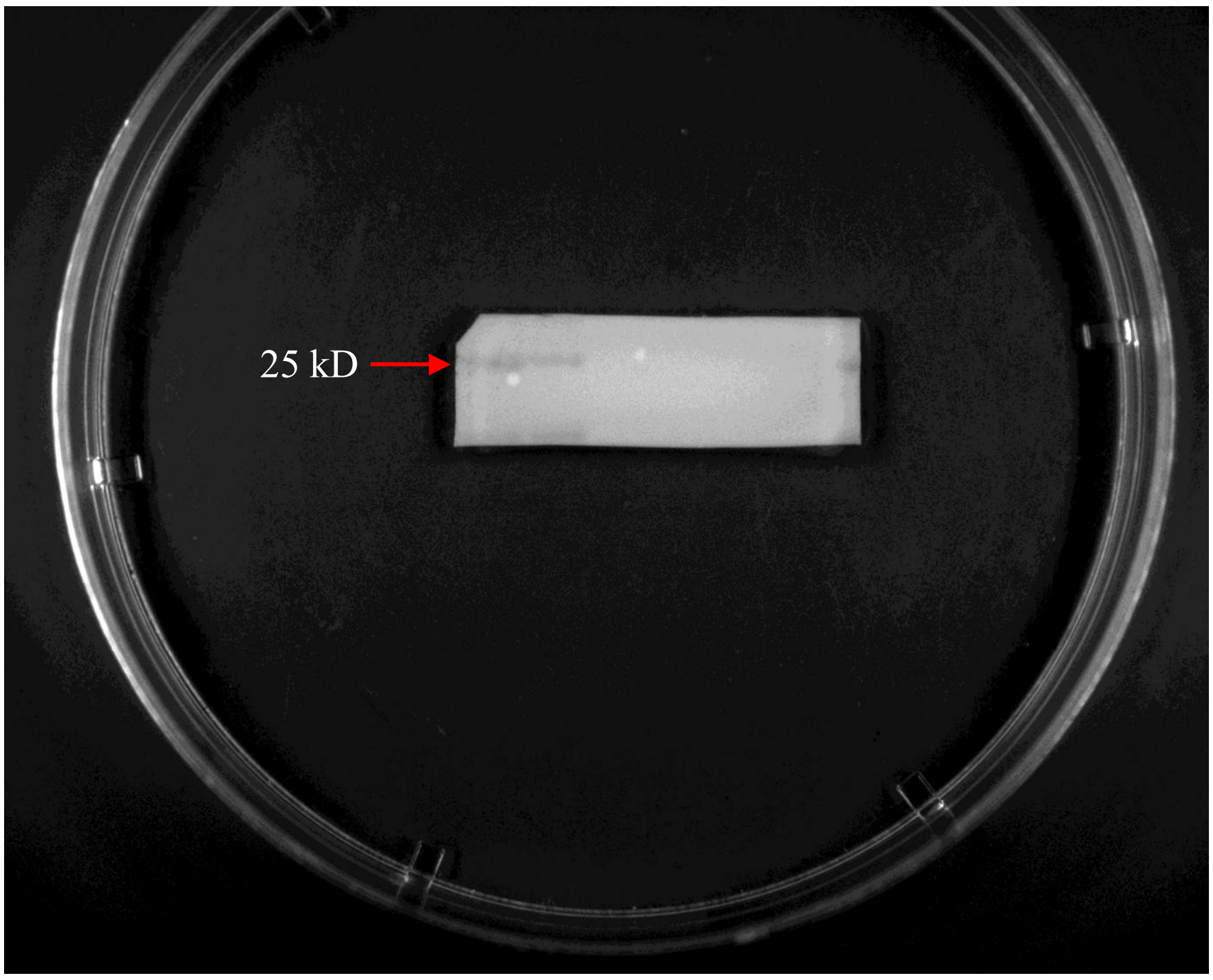

6. SOCS3-25 kDa (white)

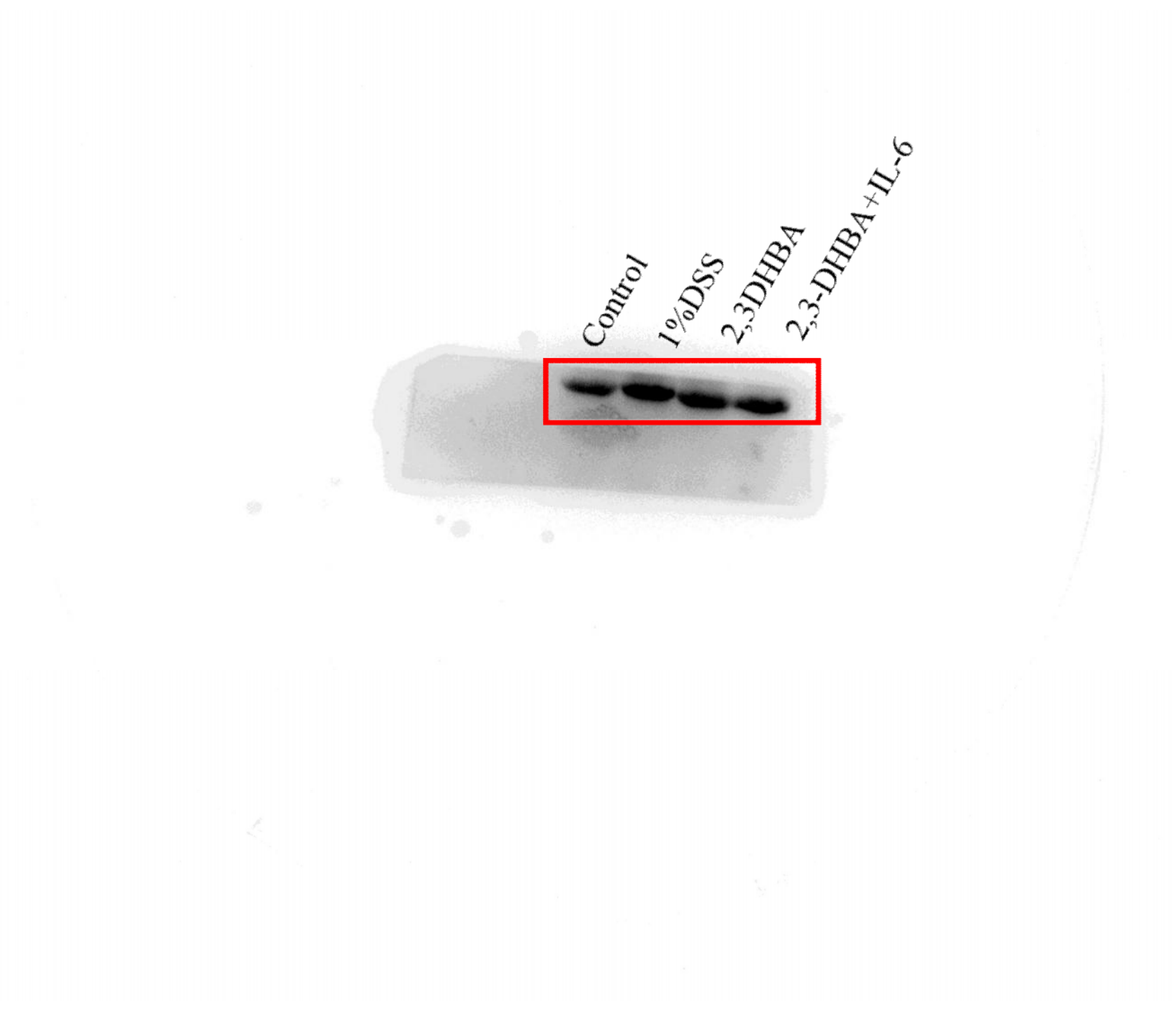

7.β-actin-43 kDa (chemi)

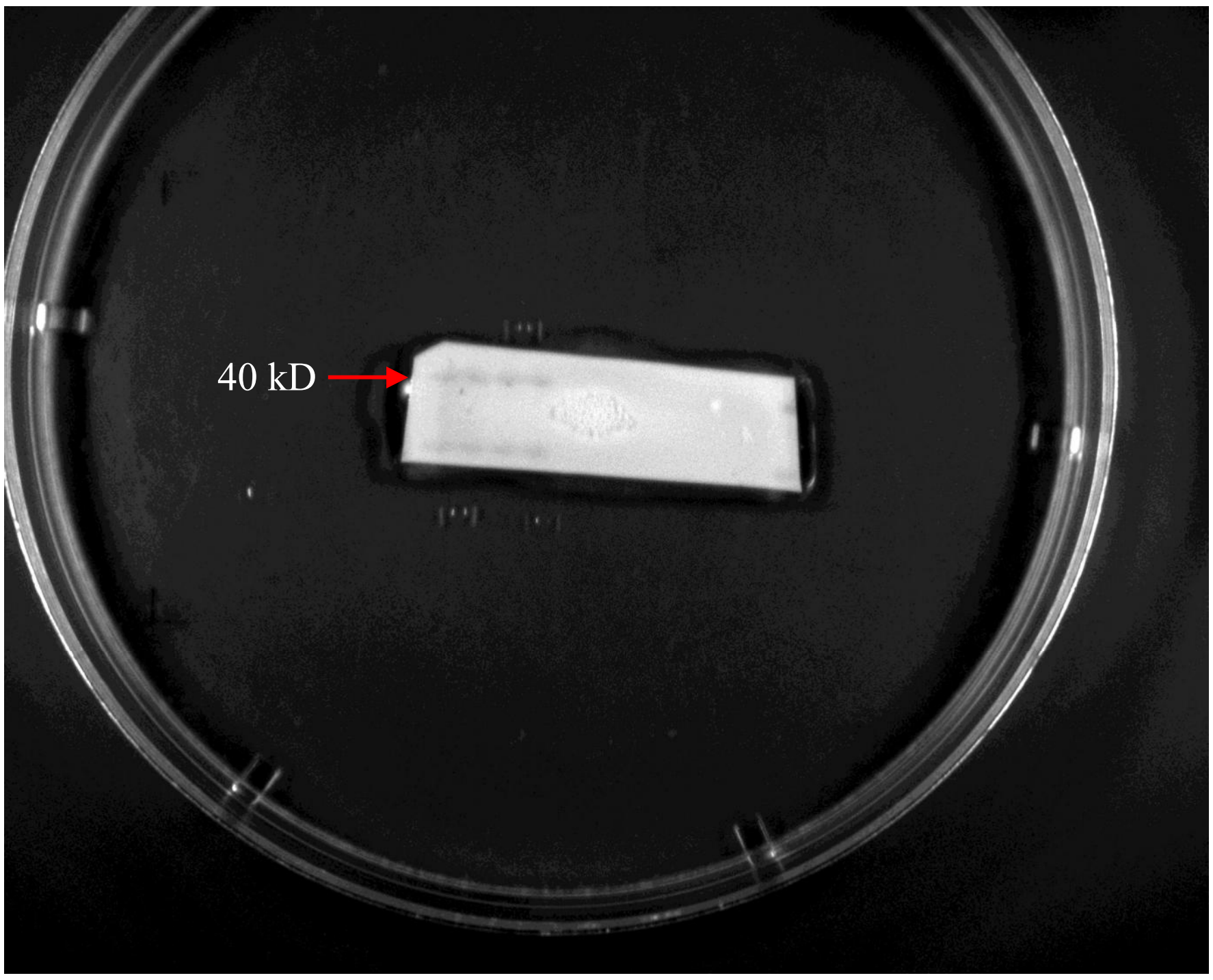

7.β-actin-43 kDa (white)
